# A recent and rapid genome expansion driven by the amplification of transposable elements in the Neotropical annual killifish *Garcialebias charrua*

**DOI:** 10.1186/s40659-025-00649-8

**Published:** 2025-11-27

**Authors:** Felipe Gajardo-Escobar, Camilo Valdivieso, Alex Di Genova, Luisa Pereiro, Maria Jose Arezo, Gino Nardocci, Natalia Rojas, Verónica Gutiérrez, Nicolás G. Papa, Nibia Berois, Alex Orellana, Rodrigo A. Gutiérrez, Mauricio González, Marco A. Mendez, Martín Montecino, Christian Hodar, Alvaro Glavic, Alejandro Maass, Graciela García, Miguel L. Allende

**Affiliations:** 1 Millennium Institute Center for Genome Regulation, Universidad de Chile, Las Palmeras 3425, Santiago, Chile; 2 Facultad de Ciencias, Universidad de Chile, Las Palmeras 3425, Santiago, Chile; 3 Mathomics, Center for Mathematical Modeling (CMM), Universidad de Chile, Beauchef 851, 7th Floor, Santiago, Chile; 4 Laboratorio de Biología Molecular de Organismos Acuáticos, Sección Biología Celular, Facultad de Ciencias, Universidad de la República, Iguá 4225, Montevideo, Uruguay; 5 Faculty of Biological Sciences and Faculty of Medicine, Center for Biomedical Research, Universidad Andres Bello, Santiago, Chile; 6 Laboratorio de Biología Molecular de Organismos Acuáticos, Sección Genética Evolutiva, Facultad de Ciencias, Universidad de la República, Iguá 4225, 11400 Montevideo, Uruguay; 7 Centro de Biotecnología Vegetal, Facultad de Ciencias Biológicas, Universidad Andrés Bello, 8370146 Santiago, RM Chile; 8 Departamento de Genética Molecular y Microbiología, Facultad de Ciencias Biológicas, Pontificia Universidad Católica de Chile, Santiago, Chile; 9 Millennium Nucleus Center for Plant Systems and Synthetic Biology, Pontificia Universidad Católica, Santiago, Chile; 10 Present Address: Laboratorio de Epigenética, Pontificia Universidad Católica, Santiago, Chile; 11 Laboratorio de Bioinformática y Expresión Génica, Instituto de Nutrición y Tecnología de los Alimentos (INTA), Universidad de Chile, El Líbano 5524, Macul, Santiago, Chile; 12 Departamento de Ingeniería Matemática, Universidad de Chile, Santiago, Chile; 13 Present Address: Departamento de Biodiversidad y Genética, Instituto de Investigaciones Biológicas Clemente Estable, Avenida Italia 3318, 11600 Montevideo, Uruguay; 14 Present Address: Laboratorio de Bioinformática y Bioestadística del Genoma, Instituto de Nutrición y Tecnología de los Alimentos (INTA), Universidad de Chile, Santiago, Chile

**Keywords:** Annual killifish, *Garcialebias*, Transposable elements, Genome expansion

## Abstract

**Background:**

Neotropical annual killifish survive in seasonal ponds due to their ability to undergo embryonic diapauses in the dry season and grow, reproduce and die in the span of a few months during the rainy season. The *Austrolebias* genus group is endemic to the South American basins and shows remarkable speciation and genetic plasticity. Within this fish group *Garcialebias charrua* is sympatric with another annual killifish, *Cynopoecilus melanotaenia,* belonging to a tribe that diverged about 25 million years ago. Despite being closely related species within the Rivulidae family, both species show important differences in genome size. Here, we explore the genomic structure of these species to understand their evolution and unique adaptations.

**Results:**

We have sequenced the genomes of *G. charrua* and *C. melanotaenia* and determined that they show important structural differences between them. While *C. melaotaenia* has a genome size of around 1 Gb, similar to that of most characterized teleosts, *G. charrua* has undergone an evolutionarily recent and massive genome expansion, with a size three times larger (3 Gb). The expansion of the genome in *G. charrua* has occurred due to amplification of repetitive elements, most recently from the LINE class of elements. We explore and characterize in detail the contribution to genome expansion of repetitive elements at the level of superfamilies, as well as analyze the relationship between these elements and coding genes in *G. charrua*. We also examine the selection pressures on gene sequences and identify functions that are under positive or purifying selection, and compare these data with that derived from other species.

**Conclusions:**

Our study adds a crucial element to the understanding of annual fish evolution and life history. We show that the genetic variability and plasticity in *G. charrua* is accompanied by a recent genome-wide expansion with an important contribution of repetitive elements. By comparing these findings with data from other species, we show that *G. charrua* has undergone bursts of repetitive element expansion, with specific superfamilies of retrotransposons and DNA transposons being the most prevalent and recent. In addition, we characterize genes that are potentially implicated in adaptive traits because of their interaction with mobile elements or because they display evidence of intensified selection. These genes are candidates for functional studies aimed at unraveling the genetic basis of annualism.

**Supplementary Information:**

The online version contains supplementary material available at 10.1186/s40659-025-00649-8.

## Background

Annual killifish are Cyprinodontiformes teleosts that can survive in habitats that change seasonably, where the ponds they inhabit dry out during part of the year. They have evolved unique adaptations such as a short lifespan and embryos that undergo diapause, a reversible arrest of embryogenesis that allows the animals to resist desiccation [1]. In killifish, the annual lifestyle has likely arisen more than once as there are Old and New World annual fish that have these features [2]. In the Neotropics, the tribe Cynolebiini is one of the main groups of annual killifish, emerging about 17 Mya in Eastern Brazil [3]. A particularly diverse group within the Cynolebiini is the *Austrolebias *sensu lato, which includes at least 52 species endemic to Argentina, Uruguay and southern Brazil [4–6]. Interestingly, species of the *Austrolebias* genus group have originated from an allopatric radiation event, occurring as recently as during the mid to late Miocene (11–12 Mya) [7, 8]. The species *Garcialebias charrua* is endemic to Uruguay and southern Brazil [8, 9], as other members of this genus group, lives in temporary small ponds that dry out during the dry season. *Garcialebias charrua* is sympatric with another, widely distributed, annual killifish, *Cynopoecilus melanotaenia*, which belongs to the sister tribe Cynopoecillini*,* a group that diverged from the Cynolebiini during the Oligocene (> 25 Mya) [7]. The last common ancestor of both species inhabited Southern Brazil, in coastal plains that are now covered by the Atlantic Forest [7]. *Cynopoecilus melanotaenia* originated in the early Miocene (15–18 Mya) and, like some of its close relatives, shows significant differences with other Cyprinodontiformes, among them, internal fertilization [9, 10].

**Fig. 1:**
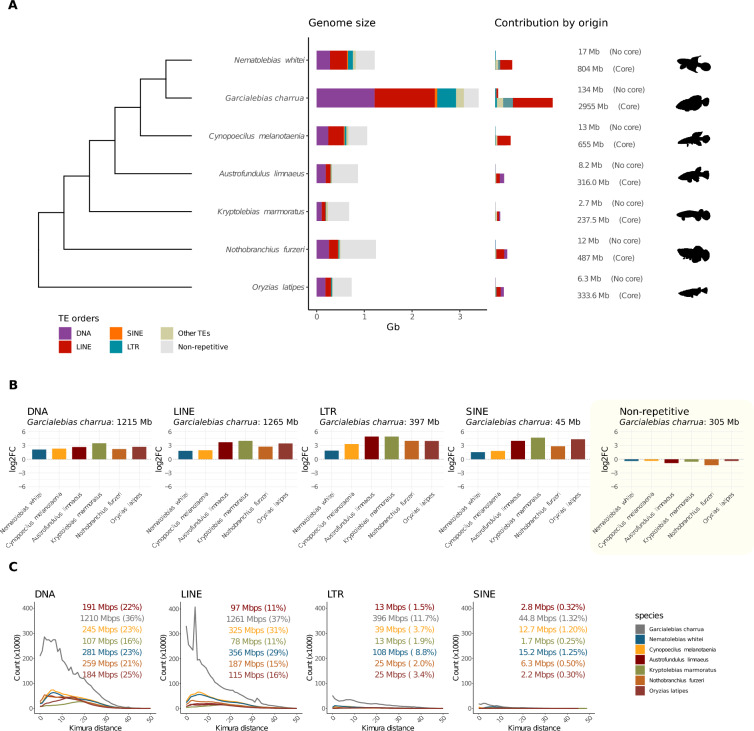
Specific orders of transposons contribute the most to the increased genome size. **A** Contribution of the most abundant TE orders to the genome size of 6 species of killifishes and the outgroup taxon (left). Representation of the proportion of bases from core and non-core superfamilies (right). **B** Log2 fold-change ratio of the abundance of the most abundant TE orders in *G. charrua* compared with the other species. **C** Distribution of Kimura distances for the most abundant TE orders in *G. charrua* compared with the other species, reflecting the divergence of TE sequences relative to their consensus

Previous work [7] has shown that members of the *Austrolebias genus* group have a genome size that is significantly larger than that of other members of the Cyprinodontiformes; while the estimated genome size of *G. charrua* is approximately 3 Gbp, *C. melanotaenia* has a genome on the order of 1 Gbp (see Fig. 1A). Importantly, *Austrolebias* genus group taxa are true diploids suggesting that their genomes have undergone this remarkable expansion likely due to repetitive sequence amplification [7, 8]. Further, given that more ancestral lineages and its sister genus *Cynopoecilus* displays genome sizes around 1 Gb, It reasonable to think that amplification occurred in the early evolution of the *Austrolebias* genus group clade, an event followed by -or concomitant with- a speciation burst [8].

Transposable elements (TEs) have been shown to be involved in the amplification of the genome size in multiple taxa [11–13]. These elements are diverse and are present in the genome of many -perhaps, nearly all- living organisms [14]. They owe their name to their ability to transpose from one location to another in the genome [15], a feature that can produce neutral, beneficial or deleterious consequences [16]. Hence, they are commonly found in heterochromatic regions, silenced by epigenetic mechanisms, and only a few of them become co-opted or domesticated [17]. TEs are classified into two main classes based on their molecular mechanisms of transposition. Class I retrotransposons require an RNA intermediate to mobilize, and include the Long Interspersed Nuclear Elements (LINE), Short Interspersed Nuclear Elements (SINE) and Long Terminal Repeat (LTR) orders. On the other hand, Class II transposons correspond to DNA transposons, in which transposition occurs in the form of DNA, without an RNA intermediate. Further functional classifications reveal that there is a wide diversity of superfamilies within each one of these orders [18].

Here, we obtain the genome sequences of *G. charrua* (3.39 Gb) and *C. melanotaenia* (1.06 Gb), two South American annual Cyprinodontiformes fish that inhabit the same environment. Using the information retrieved from the genomes of both species, together with other sequenced Neotropical and Old World killifish (and the beloniform *Oryzias latipes* as an outgroup), we performed a comparative analysis focused on the contribution of TEs to genome size and their activity in recent evolutionary time.

Additionally, it has been widely suggested that TEs contribute to shaping genome structure by furnishing peculiar environmental adaptations [19–22]. Cui et al. [23] described the genomic basis underlying adaptations to seasonal habitat desiccation in African killifishes, identifying the genetic variants associated with positive and relaxed purifying selection. In this work, we performed an analogous analysis to test this hypothesis including Neotropical killifishes (*Nematolebias whitei*, *Austrofundulus limnaeus*, and *Kryptolebias marmoratus*), the Old World annual fish *Nothobranchius furzeri,* and a member of the Beloniformes order (*Oryzias latipes*) as an outgroup, to reveal different selective constraints operating on the *G. charrua* genome.

Finally, we explored the patterns of TE insertion in the genome of *G. charrua* with focus on protein-coding genes and performed a functional enrichment analysis to gain insights into the possible functional implications of such a TE-mediated genome expansion.

## Methods

### Animal samples for DNA and RNA sequencing

All experimental procedures were approved by the Facultad de Ciencias Universidad de la República (Uruguay) Institutional Animal Care and Use Committee (IACUC; Protocol codes 240,011-002308-14 and 240,011-001192-16). The animals were captured under permission from the IACUC of the Universidad de La República in Uruguay. The animals were under no ownership or protection and, therefore, we did not require any type of consent or permission. Adult *G. charrua* and *C. melanotaenia* individuals were collected in the wild from their natural habitats (33°15′37″S, 53°53′29″W, “La Charqueada”, Treinta y Tres Department, Uruguay) and sacrificed by immersion in a solution 0.2% (w/v) 2-Phenoxyethanol. Fixed individuals and tissues were preserved in RNAlater solution (Life Technologies, USA) and kept at − 80 °C until samples were used.

### Nucleic acid extraction and sequencing

DNA was extracted from a single male individual of each species for genome sequencing using the DNeasy Blood & Tissue kit (QIAGEN, USA), according to the manufacturer’s instructions. To avoid RNA contamination, samples were treated with RNase A (Thermo Fisher Scientific, USA). DNA elutions were kept at − 20 °C until they were delivered for sequencing.

Genomic DNA was sequenced at Macrogen Inc. facilities (Seoul, Korea) in the Illumina Hiseq2500 platform (1 lane 2 × 150 bp, 2 lanes 2 × 150 bp 280 bp insert size selection and 1 lane 2 × 150 5kbp insert size selection) and 1 run in PacBio platform (15–20kbp) at the DUGSIM (Durham, NC) facilities.

Total RNA was extracted from dissected tissues (skin, muscle, testicles, eye and gill) from individuals of each species for sequencing using the RNeasy Mini kit (QIAGEN) according to the manufacturer’s instructions. DNase digestion was performed using the RNase-Free DNase set (QIAGEN) as suggested in the RNA isolation protocol. Total RNA was precipitated with 3 M sodium acetate and 100% ethanol and delivered for sequencing. RNA-Seq data was generated at Macrogen Inc., from 2 lanes 2 × 150 bp in Illumina Hiseq2500. Total RNA samples were enriched for coding transcripts with oligodT primers before the sequencing runs by the same company.

### Genome assembly

For the *G. charrua* genome assembly, we corrected the PacBio long reads using high-quality Illumina reads with the Proovread software [24] and performed an iterative assembly by simulating short read libraries with insert sizes of 1, 2, 3, 5, 7, 10, 15, and 20 kb. This strategy allowed us to extract positional information from long reads with higher error rates, while still considering high-quality regions. In the case of *C. melanotaenia*, the genome assembly was done using only paired Illumina reads. Finally, both genomes were assembled using ALLPATHS-LG (R43019) [25].

### Annotation of genes and repetitive elements

In order to obtain reliable gene annotations for the assemblies of *G. charrua* and *C. melanotaenia*, first, we trained the Augustus gene predictor [26] with a set of bonafide gene models, and applied it to the genome assemblies, excluding repetitive regions, and obtaining ab initio predictions for both species. Second, we used tblastn to identify those coding sequences matching known proteins in the Uniprot and Swissprot databases. Third, we generated transcriptome assemblies from RNA-seq data of different tissues of *G. charrua* and *C. melanotaenia* (muscle, gill, eye, skin, ovary, and testis), and then mapped the obtained transcripts to their corresponding genome assemblies to infer their original locus. Finally, we used EVM to integrate all this evidence and generate the final consensus annotation for both species.

For the identification of novel families of TEs, we performed de novo predictions using RepeatModeler [27] on the genome of each species. As several of the predicted families were annotated as unknown, we generated a complementary classification using the TERL [28] classifier, which implements deep-learning approaches. Finally, we used RepeatMasker [29] to annotate all the fragments of TEs in the genome of every species considered in this study.

### Identification of gene ortholog groups

In order to assign gene annotations to ortholog groups on each species, we used the pantherscore.pl script (version 2.2) from the PANTHER knowledgebase [30]. UpSet plots were generated using an R custom script. Alternatively, some analyses were performed using the set of ortholog groups identified by BUSCO [31].

### Quantification and comparison of TE content

We quantified the total number of base pairs (bps) of each TE order and superfamily in every species of our panel (that is, *Garcialebias charrua*, *Nematolebias whitei*, *Cynopoecilus melanotaenia*, *Austrofundulus limnaeus*, *Kryptolebias marmoratus*, *Nothobranchius furzeri*, and *Oryzias latipes*) by adding the size of the fragments identified by RepeatMasker at the corresponding level of classification. Besides, we calculated the relative contribution of each TE superfamily in regard to the size of the species genome, based on the size of their genome assemblies, and generated multiple visualizations breaking down the contribution of every TE superfamily to the genome size (See the “Availability of data and materials” section).

In order to gain a deeper understanding of the magnitude of the expansion of TEs in the genome of *G. charrua*, we compared the absolute abundance of the TE orders and superfamilies in this species with the analogous abundances in the rest of the panel. For this, we calculated the Log_2_ fold-change of the absolute number of bps associated with the most abundant TE orders and superfamilies in *G. charrua*, over the corresponding value in each one of the other species, as follows:

LogFC_bps_ = Log_2_(Number of bps in *G. charrua* for the TE superfamily T/Number of bps in specie S for the TE superfamily T).

Regarding the analysis of shared and unique TE superfamilies, we used the classification of TEs to infer the presence or absence of every superfamily in the genome of the 7 species evaluated in this work. Thus, we could define a core set of TE superfamilies present in all the species, which we focused on given their significant contribution to the genome size of all species.

Finally, for all the methodology of this section and the visualization of results, we developed multiple R scripts and workflows which we organized and documented into a single repository (See the “Availability of data and materials” section).

### Analysis of overlapping between TEs and genes

We calculated the number of fragments overlapping gene regions in *G. charrua* using a custom R script developed using the GenomicRanges library (See the “Availability of data and materials” section) [32]. The strategy was, first to obtain all the fragments for each TE superfamily of interest, and merge redundant annotations caused by overlapping fragments of TEs using the “reduce” function. Next, we calculated the overlapping of this non-redundant set of TE fragments with genes, irrespective of their location within them. This allowed us to calculate the number of TE fragments and percentage of the gene length occupied by them for each one of the superfamilies evaluated. In parallel, we performed the same strategy on exons, introns, and a 500 bp flanking region upstream and downstream of the gene boundaries, aimed to recover 5′- and 3′-UTRs, respectively.

Additionally, we extracted the nucleotide sequence of all overlapping TE regions longer than 500 bps and 1 kb and then used the TRF program with the recommended parameters (“2 5 7 80 10 50 2000 -l 10 -h -ngs”) to predict tandem repeats [33]. Finally, we performed ranked and unranked enrichment analyses of the set of genes having tandem repeats using the “enricher” and “GSEA” functions, respectively (both from the clusterProfiler R package) [34]. For this, we defined two sets of genes, those containing any TE region predicted to contain tandem repeats; and those containing TE regions with a number of tandems over the 99% quantile of the whole set of identified tandem regions.

### Analysis of neutral or intensified selection

We restricted the analysis to all single-copy genes shared by the 7 species studied in this work, based on the set of conserved genes identified by BUSCO [31]. Thus, we defined a set of ortholog groups of single-copy genes. Then, we used Clustal Omega [35] to perform multiple sequence alignments (MSA) of the aminoacid sequences corresponding to these genes and used Pal2nal [36] to obtain the analogous “codon-aware” nucleotide alignment. In parallel, we used RAxML [37] with the original amino acid alignments, 100 bootstrap replicates, and the PROTGAMMAGTR model to generate one phylogenetic tree for each ortholog group, which together with the corresponding “codon-aware” nucleotide alignments served as input for RELAX, a method of the HyPhy suite for assessing the degree of the intensity (or relaxation) of selection [38].

### Phylogenetic analysis of representative sequences of TEs across species

First, we filtered all the fragments of TEs previously identified with RepeatMasker, maintaining those with a fragment size higher than 1 kb, and belonging to one of the 6 superfamilies that we found to be the most numerous in *G. charrua,* that is, L2, Rex-Babar, RTE-BovB, TcMar-Tc1, hAT-Ac, and hAT-Charlie. Next, we ran a clustering analysis using CD-HIT [39], in order to identify representative nucleotide sequences (centroids). For this, we considered 70% of sequence identity as a cutoff for a TE fragment to belong to a cluster, each one being represented by its longest fragment, the centroid. We continued our analyses using all the identified centroids as a means to dimensionality reduction of the repetitive content of the genomes. Next, we aligned these sequences using Clustal Omega [35] with the “–use-kimura” parameter and filtered the alignments keeping only those centroids with an alignment coverage higher than a given threshold. Note that, because of differences in the number and heterogeneity of fragments among TE superfamilies, we set a specific alignment coverage for each one of them, for instance, for L2 (80%), Rex-Babar (30%), RTE-BovB (60%), TcMar-Tc1, hAT-Ac (30%), and hAT-charlie (30%). Finally, we used RAxML [37] for maximum likelihood phylogenetic inference using 100 bootstrap replicates, and the GTRCAT and GTRGAMMA substitution models for DNA transposons and retrotransposons, respectively. Tree visualizations were generated using the R scripts contained in the TE-workflows repository (See the “Availability of data and materials” section).

## Results

### Genome assembly and gene annotation

We extracted and sequenced genomic DNA from one individual of *G. charrua* and one of *C. melanotaenia*, obtaining a total of 195,828,591,855 bp and 94,074,164,468 bp, respectively. From this throughput we estimated a genome coverage of 65.2X for *G. charrua* and 94X for *C. melanotaenia*. Notably, for *G. charrua* four different sequencing experiments including short and long reads was not enough to achieve a coverage comparable to the one obtained in *C. melanotaenia* using only one library of short reads (Table S1). Despite this, our strategy allowed us to assemble, at scaffold level, the genomes of both species. As is shown in Table 1, *G. charrua* has a genome size of 3.39 Gbp, the largest genome among the teleosts included in this study. Furthermore, after exploring the repetitive content per species we realized that, in *G. charrua,* 76.5% of the genome is composed of repetitive elements, which far exceeded the repetitive content of the other fishes (Table 1). Additionally, we assessed the quality of the assemblies comparing the complete panel of selected species using BUSCO. Notably, despite using both long and short read sequencing strategies, we were only able to recover 67.2% and 65.8% of complete orthologs in *G. charrua* and *C. melanotaenia*, respectively. These percentages are markedly lower in comparison to the other genomes (Table 1). We hypothesized that the high repetitive content in *G. charrua* and *C. melanotaenia* pose obstacles to the challenge of generating good-quality assemblies.

**Table 1:** Comparison of genome assemblies

	*G. charrua*	*N. whitei*	*C. melanotaenia*	*A. limnaeus*	*K. marmoratus*	*N. furzeri*	*O. latipes*
Assembly size	3,393,953,585	1,218,316,901	1,058,579,302	866,963,281	680,655,669	1,242,518,059	734,057,086
Number of scaffolds	117,134	18,998	154,991	29,785	300	5,897	25
Longest scaffold	292,228	95,146,955	247,638	11,189,062	38,021,566	98,476,147	38,248,663
N50 scaffold	35,803	49,984,095	13,486	1,098,383	28,170,849	57,367,160	31,218,526
Number of contigs	261,723	92,576	164,410	158,069	25,553	72,429	516
Longest contig	152,256	330,075	128,470	133,211	688,134	193,296	10,255,628
N50 contig	16,829	27,766	11,105	9,107	61,321	20,963	2,530,934
% Repetitive	76.5	54.58	52.2	32.84	28.91	35.64	39.83
*BUSCO*							
% Complete	67.2	92.8	65.8	84.5	97.8	86.6	96.8
% Single copy	48.4	91.7	62	84.2	97.1	86.3	96.2
% Duplicated	18.8	1.1	3.8	0.3	0.7	0.3	0.6
Accession number	–	GCF_014905685.2	–	GCF_001266775.1	GCF_001649575.2	GCF_001465895.1	GCF_002234675.1
Reference	This study	Thompson et al. [40]	This study	Wagner et al. [41]	Kelley et al. [42]	Reichwald et al. [43]	Ichikawa et al. [44]

We also evaluated whether the high repetitive content found in the genome of G. charrua impacted its content of genes. A total of 40,933 genes were annotated, which is almost twice the number reported for other teleost genomes (e.g., *A. limnaeus*, *N. furzeri*, *O. latipes*; Table 2). While this elevated count may partly result from the fragmented nature of the *G. charrua* assembly, it is notable that *C. melanotaenia*, the most fragmented assembly in the dataset (N50 = 13,486), shows a number of predicted genes comparable to other species. This suggests that, although gene number may be overestimated, a genuine increase in gene content cannot be ruled out and may be linked to TE-driven expansion (see Discussion).

**Table 2:** Comparison of gene annotations

	*G. charrua*	*N. whitei*	*C. melanotaenia*	*A. limnaeus*	*K. marmoratus*	*N. furzeri*	*O. latipes*
Number of genes	40,933	23,454	20,236	26,626	25,206	25,049	25,943
Number of exons	247,280	260,815	146,753	425,590	631,018	508,979	686,899
Number of introns	206,347	234,512	126,517	386,078	584,159	467,985	635,428
Avg. exon length	181.68	199.01	172.04	249.56	264.61	253.68	259.57
Avg. intron length	1402.32	2625.13	941.3	2216.29	1941.58	2436.04	1884.91
Avg. number of exons per gene	6.04	11.2	7.25	15.98	25.03	20.32	26.48
Avg. number of introns per gene	5.04	10	6.25	14.5	23.18	18.68	24.49

An intriguing observation derived from the genome data is that there is a decreased exon length in the group composed of *G. charrua*, *N. whitei*, and *C. melanotaenia* in comparison with the other species (Table 2). However, other factors such as the assembly fragmentation and high rates of duplicate genes in *G. charrua* and *C. melanotaenia* may be biasing this result. In order to exclude this possibility, we calculated the same metrics using a set of complete single-copy genes, based on the results of BUSCO (Table S2). Examination of the results showed, in this case, similar trends in exon and intron lengths in all species. Therefore, at least in the case of single-copy genes, a general trend towards shorter genes in some species can be disregarded, although this remains an open possibility in the case of duplicated genes.

### The genome size expansion of *Garcialebias charrua* is due to evolutionarily recent TE activity

All of the fish species included in this study belong to the Cyprinodontiformes order, and more specifically to the Aplocheiloidei suborder, except for the Japanese rice fish (*Oryzias latipes*), a member of the Beloniformes order, which we used as outgroup. The phylogenetic relationships shown among them were obtained from the last reported phylogeny for this order [45]. As seen in Fig. 1A, *G. charrua* is revealed to have the largest genome among the fishes studied here, with 3.4 Gbp, more than twice the size of the genome of any of the other species in the panel. Furthermore, there is evidence of a remarkable expansion of all the orders of TEs in *G. charrua*, contrasting with a slightly decreased proportion of the non-repetitive content (Fig. 1B). The TE expansion is particularly notable for the LINE and DNA orders. In contrast, the proportion of SINE, LTR, and other TEs is very similar in almost all fishes, except in *N. whitei* and *G. charrua*, where there is a significant contribution of LTR elements to the genome size (Fig. 1C).

We next explored the diversity of superfamilies within each order of TEs in every species. After identifying and classifying all families, we observed some notable differences. First, the number of families predicted for *G. charrua* far exceeded the number predicted for the other species. Second, at the superfamily level, *G. charrua* showed the largest diversity with 63 superfamilies, contrasting with its closest relative, *N. whitei*, which with 46 superfamilies, is the least diverse species in terms of superfamilies content (Table S3).

**Fig. 2:**
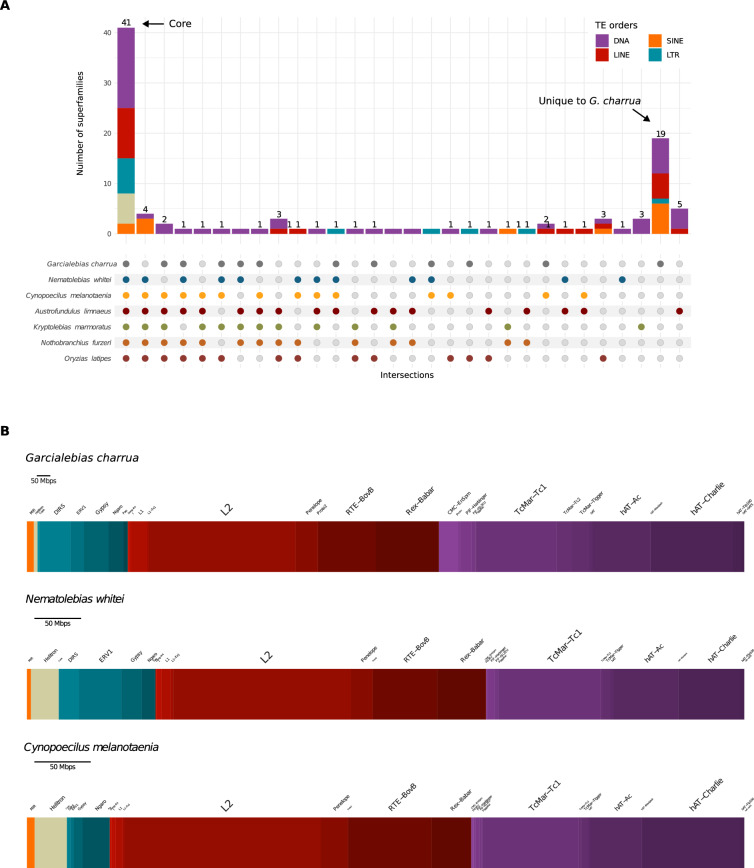
TE superfamilies shared between multiple killifishes and the outgroup species. **A** UpSet plot depicting the number of TE superfamilies shared and unique to every species. Empty intersections are not shown. **B** Contribution of TE superfamilies found in the core set relative to the genome size, calculated based on RepeatMasker annotations and normalized to assembly size

Furthermore, a core set of 41 superfamilies was identified in all fishes. Interestingly, *G. charrua* was the species with the most unique TE superfamilies, with 19 of them not found in the other fishes (Fig. 2A). Taking a closer look at this group in terms of TE content, we noted the huge contribution of the core mobile elements to *G. charrua* genome size (Fig. 1A, right). Thus, the burst in the amplification of TEs responsible for the increased genome size of *G. charrua* occurred mainly among the superfamilies shared with the other species. It is important to note, however, that this burst of expansion also seemed to affect the non-core elements, since these also showed a higher content in *G. charrua*. Hence, the genomic expansion in this annual killifish possibly was attained by an indiscriminate amplification of TEs within its genome, but with emphasis in the LINE and DNA orders.

### Specific superfamilies of retrotransposons and DNA transposons contributed the most to the increased genome size in* G. charrua*

**Fig. 3:**
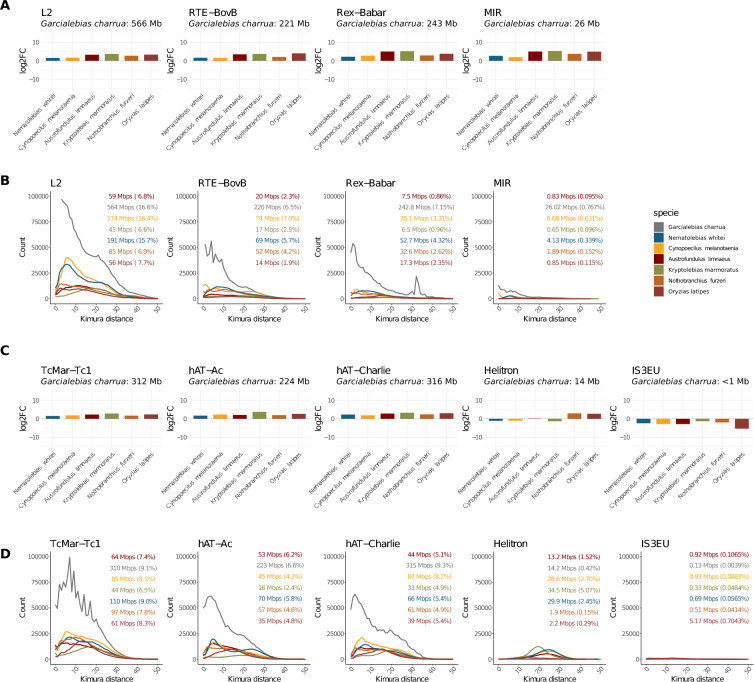
Specific superfamilies of retrotransposons and DNA transposons contribute the most to the increase in genome size in *G. charrua*. **A** Log2 fold-change ratio of the abundance of retrotransposons in *G. charrua* compared with the other species. **B** Distribution of Kimura distances for TE superfamilies of retrotransposons, showing sequence divergence relative to their consensus. **C** Log2 fold-change ratio of the abundance of DNA transposons in *G. charrua* compared with the other species. **D** Distribution of Kimura distances for TE superfamilies of DNA transposons

Our next goal was to identify and characterize the most highly represented superfamilies that contribute to the genomic expansion in *G. charrua* (See Material and Methods). To do so, a proportional barplot with the contribution of all core superfamilies was generated for *G. charrua* and its closest relatives, *N. whitei* and *C. melanotaenia* (Fig. 2B). As a result, it can be seen that the relative abundance of the shared superfamilies is similar in the three species at both the order and superfamily levels. We characterized the six most abundant superfamilies based on their contribution to the size of the genomes. These are: L2, RTE-BovB, Rex-Babar, TcMar-Tc1, hAT-Ac, and hAT-Charlie. Besides, we selected the MIR superfamily, because it is the only SINE in the core set, and the Helitron and IS3EU superfamilies, because they are the only ones with reduced abundance in the *G. charrua* genome. Then, each selected superfamily of retrotransposons and DNA transposons was examined in terms of degree of amplification in each species relative to its content in *G. charrua* (Log2FC) (Fig. 3A and C); Additionally, we assessed the sequence divergence of TE fragments by calculating their Kimura two-parameter (K2P) distances relative to the consensus sequence of the superfamily [46] (Fig. 3B and D). This metric estimates the number of nucleotide substitutions per site, accounting for differences in transition and transversion rates, thereby providing an evolutionary distance that can be used to infer the temporal divergence between sequences [46].

We found that, for the L2 LINE, the most amplified and abundant TE, there was a higher content in *G. charrua* compared to the other species. However, this difference is slightly attenuated in the phylogenetically closest species, *N. whitei* and *C. melanotaenia*. Regarding the divergence graphs, all slopes tend to increase at a Kimura value of 30, but *N. whitei* and *C. melanotaenia* show a marked increase in TE copies below that Kimura value. Furthermore, the increase observed for the slope of *G. charrua* is remarkable compared to the other fishes. Nevertheless, despite the notable difference of TE copies in *G. charrua*, the percentages reported for their contribution to genome size are quite similar between *G. charrua*, *N. whitei* and *C. melanotaenia* and, interestingly, this value is twice the contribution reported for the other species.

Another LINE selected for further analysis was Rex-Babar, where the fold-change ratios in *G. charrua* compared with the other species evidenced a pronounced difference in terms of base pair content, especially with *A. limnaeus* and *K. marmoratus.* Regarding sequence divergence among copies, only the slope of *G. charrua* is detached from those of the other fishes. Again, the start point of this divergence with the other species is approximately at a Kimura distance of 30. Furthermore, *G. charrua* shows the largest proportion of Rex-Babar contribution relative to genome size among fishes, with 7.15%. Interestingly, a high number of Rex-Babar sequences are uniquely detected at a Kimura distance of 30 for *G. charrua* (Fig. 3A).

The third LINE analyzed was RTE-BovB. For this retrotransposon, as well as for the two others described above, there are more fragments in *G. charrua* than in the other fishes. Interestingly, the Kimura distance distribution shows a remarkable difference in terms of copy number among fishes, and has a positive slope approaching a Kimura distance of 0, suggesting that the transposition events for this LINE are still ongoing. However, for RTE-BovB, the highest proportion in terms of genome size contribution is observed in *C. melanotaenia* with 7.0%, followed by *G. charrua* with 6.5%.

Taking into account the three LINE superfamilies described above, they comprise a total of 1 Gbp, approximately one third (30.25%) of the genome size of *G. charrua*. Hence, despite the significant contribution of all LINEs to genomic size, these three superfamilies, by themselves, explain a large proportion of the genome expansion in *G. charrua*.

Nonetheless, there are expansions of retrotransposons which do not contribute substantially to the genome size. For example, the MIR SINE contributes with 26 Mbps which accounts for only 0.76% of the *G. charrua* genome. Interestingly, the MIR superfamily shows a delayed activation according to a Kimura slope diverging from the other species at a distance of 20, which is concordant with SINEs relying on the transpositional machinery of LINEs (Fig. 3A and B).

Further examination of the other TE orders that contributed the most to the genome size expansion in *G. charrua* (for instance, DNA transposons), led us to select the TcMar-Tc1 superfamily. As expected, there is a larger contribution in base-pairs of this superfamily in *G. charrua* compared to the rest of the species studied. Notably, the Kimura slope of *G. charrua* shows an increased content of TcMar-Tc1 together with periodical bursts of ancestral transposition events (Fig. 3C and D).

In the case of the DNA transposon hAT-Charlie, our analysis reveals a larger TE content (315 Mbps) for *G. charrua* in comparison to the other fishes. Moreover, the fish with second largest hAT-Charlie content is *C. melanotaenia* with 87 Mbps, a remarkable difference. Taking these two species as a reference, they also possess a larger proportion of TEs in comparison to their genome size (i.e. 9.3% for *G. charrua* and 8.2% for *C. melanotaenia*). Even though *C. melanotaenia* shows a larger contribution of hAT-Charlie elements to its genome size, the *G. charrua* Kimura slope becomes evidently separated from the slopes of the other species. And again, this difference becomes obvious at Kimura 30.

Similarly, the hAT-Ac superfamily shows a larger TE content in comparison to the other species with a total of 223 Mbps contributing 6.6% of the *G. charrua* genome size. This proportion is similar to the 6.2% shown in *A. limnaeus*, but interestingly, its genome content of 53 Mbps is even smaller than the 70 Mbps accumulated in *N. whitei*. Regarding Kimura slopes, the detachment of *G. charrua* from the other fishes begins, once again, at Kimura 30.

Finally, we selected the Helitron and IS3EU superfamilies because these are the only ones which are reduced in the genome of *G. charrua*. More specifically, both of them are reduced in all the Neotropical species, however, contrary to the Helitron superfamily, IS3EU is even more reduced in *G. charrua*. These superfamilies also show particularities in their Kimura slopes. For instance, Helitron shows a gaussian-like distribution, with the highest activity at moderated Kimura distances (among 10 and 30), but that in the case of *G. charrua* are biased towards more recent evolutionary time. On the other hand, IS3EU shows a low basal activity in all species with no evident peaks at the scale of the expanded superfamilies.

### The amplification of TEs affected multiple lineages within the most abundant superfamilies

Our previous analyses showed that, with a few exceptions, the expansion of repetitive elements in *G. charrua* affected almost every TE superfamily, resulting in a global increase in the genome size of this species. However, it is not clear if the TE amplifications that resulted in the increase of genome size of *G. charrua* were associated with a specific family (lineage) of TEs. In order to evaluate if the contribution of the most abundant TE superfamily to the genome size is concentrated on some families or it is rather nonspecific, we performed a phylogenetic analysis using sequences of representative TEs from all the species evaluated in this study. We found few well-supported groups, and those with high support usually exclude TEs representing a high contribution to the genome size in *G. charrua*, suggesting that the expansion of repetitive elements took place in lineages uniquely found in *G. charrua*; this is particularly true for the L2 and hAT-Charlie superfamilies (Figs. S1 and S6).

**Fig. 4:**
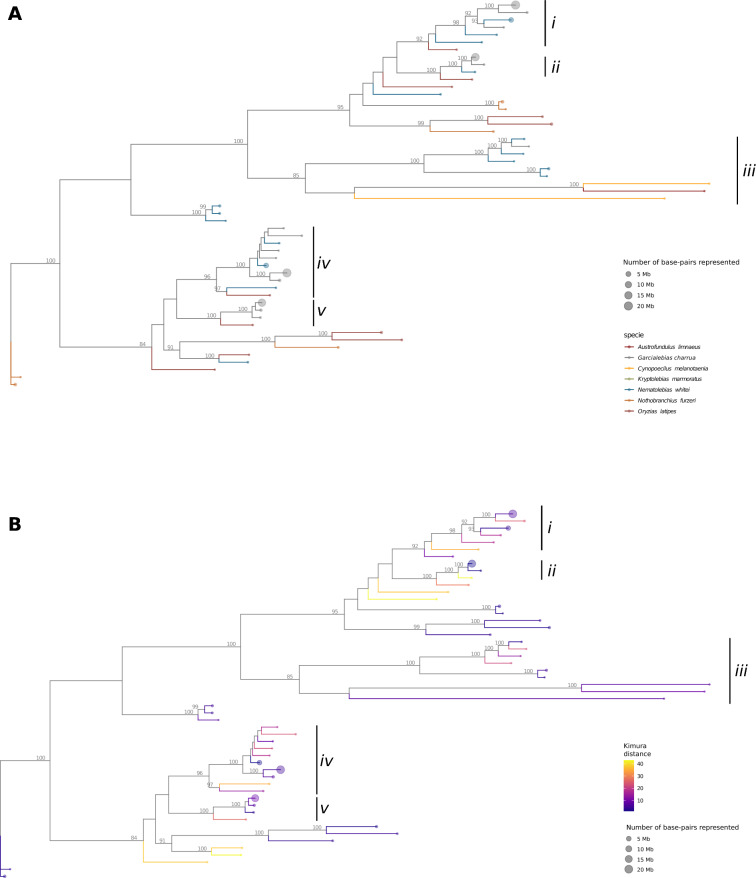
The amplification of TEs affected multiple lineages within the most abundant superfamilies. **A** Maximum likelihood tree based on sequences of representative TEs (centroids) for the Rex-Babar superfamily with bootstrap support obtained from 100 replicates. Edges are colored by species. Roman numbers indicate particular clades discussed in the main text. **B** Same tree as (**A**), but edges are colored by the Kimura distance associated with the representative TEs

Nonetheless, there are some clades that link *G. charrua* with other Neotropical species. For example, for the Rex-Babar superfamily we identified 5 well-supported clades (Fig. 4A, clades i, ii, iii, iv, and v), where 3 of them link sequences representative of TEs contributing between 15–20 Mb to the genome of *G. charrua* (clades i, ii, iv, and v). Moreover, most of the other members of the clade do not surpass the 5 Mb. Notably, the only exception occurs in its sister species, *N. whitei*, with two sequences representing TE contributions of nearly 10 Mb to its genome (clades i and iv). Similar cases are observed in the trees for the RTE-BovB (Fig. S3) and TcMar-Tc1 (Fig. S5) superfamilies.

On the other hand, when assessing the relative “age” of representative TEs in relation to their phylogenetic relationships using the Kimura distance, we found that in the Rex-Babar superfamily, the TEs representing the most expanded families in *G. charrua* show lower Kimura distances in comparison with the other species, implying a more recent origin (Fig. 4B).

Altogether, these results suggest that the contributions of TEs that explain the majority of the increase in genome size in *G. charrua* are mostly non-specific regarding the lineages within superfamilies. Moreover, in the few cases in which it was possible to establish an inter-species phylogenetic relationship between representative TEs, they do not show the same degree of expansion on their host species. This suggests that, even when some TE lineages may be shared, it is likely that their expansion is an stochastic species-specific phenomena.

### Single-copy ortholog genes are under intensified purifying selection in *G. charrua*

**Fig. 5:**
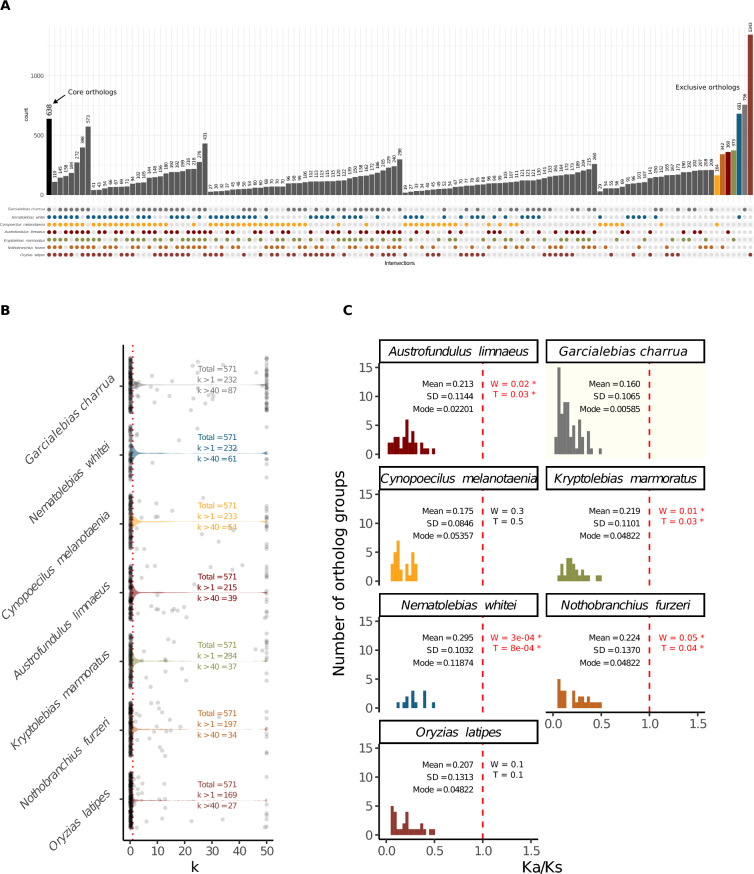
Single-copy ortholog genes are under intensified selection in *G. charrua*. **A** Shared and unique PANTHER ortholog groups. **B** Violin plots depicting the distribution of k values calculated by RELAX for each species based on 571 single-copy orthologs shared by all the species on the panel. Genes with k values higher than 1 are under intensified selection. **C** Distribution of Ka/Ks ratios for genes under intensified selection (k > 1). The red line indicates neutral selection. “W” and “T” are *p*-values from Wilcoxon and t-tests comparing G. charrua with each species. *P* < 0.05 is marked with “*”

It is known that in *Nothobranchius sp.* there is a global relaxation of selection, which correlates with a moderated increment of the genome size caused by the expansion of TEs [23]. This has not been previously assessed in Neotropical killifish and, in the former study, no metric on relaxation of selection was reported for *A. limnaeus*, the only Neotropical killifish included. In order to gain insights into this matter, we used RELAX [38] to estimate a “k” parameter, which accounts for the deviation of selection from neutrality. Thus, k values below 1 account for genes undergoing relaxed selection; while k values higher than 1 account for genes undergoing an intensification of either positive or purifying selection. For this analysis, we focused on the subset of shared single-copy genes in order to avoid the effect of paralogs, which may bias the results given the high proportion of duplicated genes found in *G. charrua* (Fig. 5A). This analysis revealed that the largest number of genes had k values lesser than 1, meaning that most shared single-copy genes are under relaxed selective constraints in all the species. However, when comparing between species, *G. charrua* has one of the largest number of single-copy genes at high values of k, thus implying an intensification of selection in this species (Fig. 5B). Indeed, when assessing the Ka/Ks ratio (Fig. S7) of genes undergoing intensified selection, we found that purifying selection is significantly stronger in *G. charrua* (Figs. 5C and S8).

### Patterns of TE insertion in gene-coding regions

It is known that TEs are not randomly inserted but, instead, they have biases regarding their insertion sites, which contributes to the generation of multiple patterns and in some cases hotspots of TE integration [47]. In order to gain insights into this matter we explored the localization of TE fragments from the most abundant superfamilies in the genome of *G. charrua* (L2, Rex-Babar, RTE-BovB, TcMar-Tc1, hAT-Ac, and hAT-Charlie), focusing on their positioning regarding genes.

**Fig. 6:**
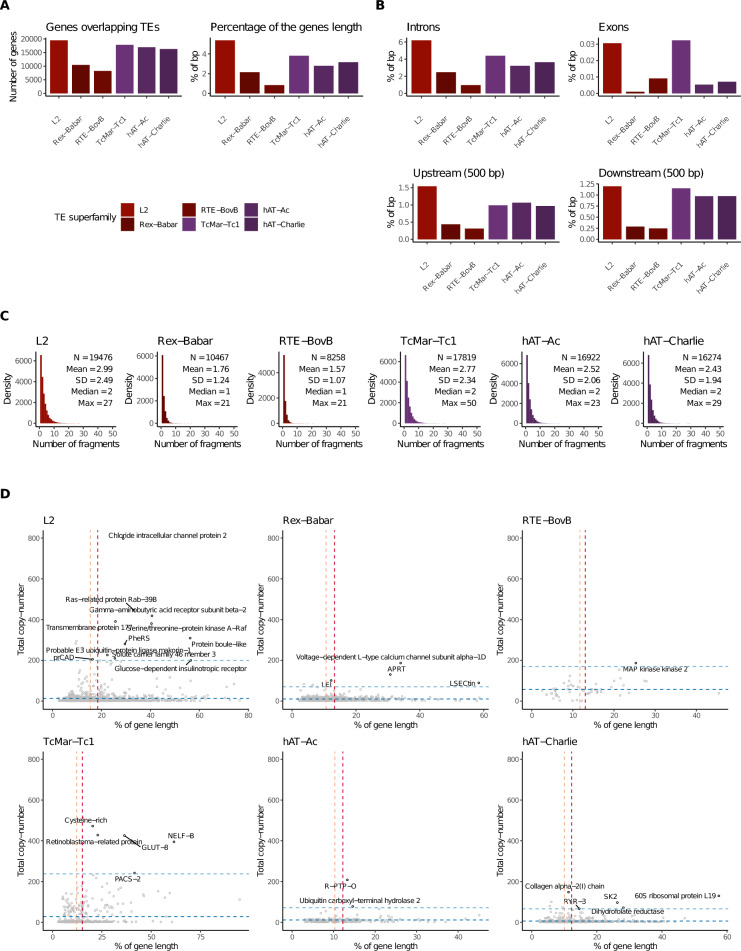
Patterns of TE insertion in genes. **A** TE-derived tandem repeats of the most abundant TE superfamilies overlapping genes and their respective contribution to the gene length. **B** TE-derived tandem repeats of the most abundant TE superfamilies overlapping gene subregions (exons, introns, UTRs). **C** Distribution of the number of TE fragments on genes for the most abundant TE superfamilies. **D** Genes with the highest content of tandem repeats derived from the most abundant TE superfamilies in *G. charrua*. Only genes with TE regions longer than 1 kb are named. Tandem repeats were predicted using TRF with recommended parameters

We found an average of 14,869.3 genes sharing the same genomic location with the selected superfamilies, with L2 and TcMar-Tc1 being the most prevalent (Fig. 6A, left), in accordance with them having the highest number of TE fragments. Moreover, the L2 superfamily is the one contributing the most to the overall gene length in comparison with all the other superfamilies assessed (Fig. 6A, right). Next, we further explored the contribution of these superfamilies to gene subregions. As expected, we found a higher proportion of TEs in introns (~ 6%) rather than in exons (~ 0.03%), upstream (~ 1.5%), and downstream regions (~ 1.25%) (Fig. 6B). Similar patterns of TE superfamily contribution to the length of all gene subregions were observed in all the other species; however, it is notable that *G. charrua* has a proportionally higher contribution of every TE superfamily to intronic regions (Figs. S9–S15).

Further, we plotted the distribution of genes in relation to their colocalization with non-contiguous TE fragments. In general, we found that all the superfamilies show a relationship similar to a power law, with the highest number of genes having a low number of fragments, and very few genes with high numbers of fragments. Besides, when comparing superfamilies, we found that there are generally more genes with a higher number of DNA transposon fragments than for retrotransposon fragments (Fig. 6C).

We also explored the content of TE regions containing tandem repeats that are within genes. We found that *G. charrua* is the species with the highest number of tandem regions in both absolute and proportional terms (Table 3). Besides, we analyzed the distribution of period sizes (the size of each tandem repetition) and found that *G. charrua* has overall shorter repetitions in comparison to most of the other species (Figs. S16 and S17). In order to assess the possible functional effects of tandem repeats in the genome of *G. charrua*, we explored genes in terms of the number of overlapping tandem repeats of each superfamily, and the contribution of these regions to overall gene length in *G. charrua*. We identified a total of 7,286 genes containing tandem repeats with an overall length of more than 500 bp; 93 of these were cataloged as “highly tandem” because of their extreme position in relation to the complete copy-number distribution (above the 99th percentile) (Fig. 6D). Then, we performed ranked and unranked functional enrichment analyses on these two gene sets. In the unranked approach, we were able to identify the “exosome multienzyme ribonuclease complex” (GO:0000178) category as enriched when evaluating the “highly tandem” set using the complete set of genes of *G. charrua* as background set (Adj. *P*-value = 0.037). On the other hand, when ranking by copy-number of tandem repeats, we found the “catalytic activity” (GO:0003824) functional category enriched on the “highly tandem” set using both the complete gene annotation of *G. charrua* (Adj. *P*-value = 0.00229), and the tandem containing sets (Adj. *P*-value = 0.00376) as background. Finally, when ranking by contribution to gene length, we found two significant functional categories enriched when using the complete gene annotation as background set. Those are the “intracellular anatomical structure” (GO:0005622) and “transcription cis-regulatory region binding” (GO:0000976) categories (Adj. *P*-value = 0.000131, and Adj. *P*-value = 0.023962, respectively).

**Table 3:** Summary of the identification of TE-derived tandem repeats

	*G. charrua*	*N. whitei*	*C. melanotaenia*	*A. limnaeus*	*K. marmoratus*	*N. furzeri*	*O. latipes*
Total number of TE regions overlapping genes	221,208	1153	96,503	547,536	546,051	706,376	642,357
Number of TE regions longer than 500 bp	31,038	136	8,921	42,516	40,245	88,417	64,313
Number of TE regions longer than 500 bp with tandem repeats	9,999	38	2,502	8,082	5701	9957	8442
% of TE regions longer than 500 bp	14.03	11.80	9.24	7.76	7.37	12.52	10.01
% of TE regions longer than 500 bp with tandem repeats	32.22	27.94	28.05	19.01	14.17	11.26	13.13

## Discussion

### TE expansion is a shared feature among phylogenetically related taxa

The tree topology based on 902 orthologous genes [45] in Fig. 1A (left) reveals that the increase in TE superfamilies (Fig. 1A, center and right) is shared, to different degrees, by various genera of South American annual killifish, several Old World annual killifish species and even in a non-annual fish belonging to the sister order Beloniformes. In fact, this selected tree topology is concordant with a previous phylogenetic tree based on morphological data where *Garcialebias charrua*, *Nematolebias whitei* and *Cynopoecilus melanotaenia* are the most phylogenetically related taxa [47]. In this sense, specific superfamilies of retrotransposons and DNA transposons contribute the most to the increased genome size of two related species, *G. charrua* and *N. whitei,* and to a lesser extent, *C. melanotaenia*. This could be an indication that TE expansion follows characteristic genomic patterns ever since the existence of the common ancestor of these highly related taxa. Despite these similarities, *G. charrua* has an unusually large diploid genome, having more than twice the size of the other analyzed species (3.39 Gbp). Moreover, 76.5% of its genome is composed of repetitive elements which far exceeds the repetitive content of other reported fishes. Therefore, this species represents an exceptionally large genome for a vertebrate, being so far, the largest among diploid members of the Actinopterygii, and in particular, among annual killifish that have been successfully sequenced and assembled.

Our analyses indicate that *G. charrua* also has the largest diversity of TEs superfamilies among related species and that there is evidence of a remarkable expansion of all the orders of TEs, contrasting with the slightly decreased proportion of the non-repetitive content. Strikingly, our study also indicates that part of the TE expansion burst that increased the genome size of *G. charrua* is not specific to a single lineage or family of TEs, but that it is, rather, a global phenomenon that affects elements mainly from the LINE and DNA orders. This specific expansion is not observed in closely related species. The repetitive elements that stand out as the main genome size contributors are the L2 and hAT-Charlie superfamilies. Further, just three LINE superfamilies account for a total of 1 Gbp (30.25%), approximately a third of the genome size of *G. charrua*, supporting their contribution to the large genome expansion in this taxon. In particular, the superfamily Rex-Babar presents the largest proportion among fishes in terms of genome size contribution with 7.15%.

Oggenfuss and Croll [48] proposed that individual TE families can experience multiple distinct burst events and generate many nearly identical copies. Interestingly, among the three most abundant LINE superfamilies, the RTE-BovB retrotransposon in *G. charrua,* presents a positive slope near Kimura 0, suggesting that the transposition events for this LINE are ongoing. Of the other major orders of TEs that contribute to genome size increase, the TE superfamily TcMar-Tc1 presents a Kimura slope in *G. charrua* that evidences an increased TE content by means of multiple small bursts of transposition events at different times. Burst events can cause a local accumulation of TEs in the chromosomes generating extensive clusters of them [49]. According to these authors, events of this type are more frequent in germ cells due to the temporary relaxation of the epigenetic control of TEs during early development. In fact, diapauses in annual fishes, such as those analyzed here, could represent an extended temporal window allowing TEs to avoid the selective constraints that otherwise restrict their activity and would allow them to propagate in the host genome.

### TE insertion and genome structure dynamics in the *Austrolebias* genus group

The genomic distribution of TEs has been associated with nuclear architecture [11]. Possible events of genomic instability at the cytogenetic level as a by-product of TE insertion bursts have been detected in the *Garcialebias* genus as it was suggested for most species of the *Austrolebias* genus group [50, 51]. These earlier studies showed extensive variation in chromosome numbers, and in the number of chromosome arms in each species, involving both types of Robertsonian (centric fusions) and non-Robertsonian (pericentric inversions) chromosome rearrangements occurring at intra- and interspecific levels. At present, it is well accepted that the insertion of TEs can induce chromosomal rearrangements as a by-product of transposition events, promoting genomic structural variation [52, 53]. In humans, the high abundance of LINE-1 and Alu repeats (approximately 17% and 11% of the human genome, respectively) favors ectopic recombination or recombination between non-homologous loci. Such recombination often results in significant chromosomal rearrangements such as gene deletions, duplications, translocations or chromosomal inversions [54–56]. Some authors, [52, 57–59] proposed that the majority of the TEs are located in heterochromatic regions. LINEs are enriched in AT-rich and gene-poor regions in the genome and in heterochromatin [11]. Since recombination is often suppressed in these regions it could explain why these chromosome segments are prone to the accumulation of TEs, where unequal crossing-over or genetic drift are common events. In the *Austrolebias* genus group, the C-banding technique detected constitutive heterochromatic blocks distributed at centromeric, telomeric, and interstitial regions following characteristic and variable patterns among species. These findings were interpreted as a result of pericentric inversions and centric fusions occurring alternatively during extensive karyotypic reorganization in cladogenetic events within this group [50, 51, 60].

Additionally, Wells and Feschotte [61] revisited the classic example of several families of LINEs showing a target ribosomal RNA gene array. Such high copy-number genes offer an excellent niche for TEs because insertion in one or a few of the genes is unlikely to have immediate deleterious consequences, and TEs can be progressively purged out by recombination within the array. Studies in species of the *Austrolebias* genus group using the silver stain NOR technique to identify rDNA active regions at the cytogenetic level, revealed a high number (3–6) of them at different chromosomal positions and patterns, which was also interpreted as a by-product of chromosomal rearrangements [50, 51, 60]. In agreement with the high level of interspecific karyotypic variation, chromosome polymorphisms presenting four different cytotypes were found in individuals of *G. charrua*, belonging to ponds from the southern Laguna Merin basin [62]. The chromosome polymorphism in this taxon involves one to three chromosome pairs of bi-armed elements, where pericentric inversions involved the shift from acrocentric to metacentric chromosome type, as well as the addition/deletion of heterochromatic blocks, and NOR bearing regions. All of the extensive variation and genomic turnover found in these taxa, suggest the existence of intrinsic factors, including TE activity. As a conclusion from the present work, it is possible to re-interpret previous assumptions. For instance, the high variability detected by means of structural chromosomal changes as well as in the heterochromatin and rDNA regions distribution within and among species in the *Austrolebias* genus group, could be associated, to multiple events of TEs insertion and/or accumulation of TE tandem repeats in the aforementioned chromosomal regions. Indeed, TEs are known to generate novel repetitive sequences such as tandem repeats [63]. Interestingly, Ahmed and Liang (2012), considered that the ability of TEs to contribute to genome expansion is due, not only to transposition events, but also by generating tandem repeats producing great genome instability [64]. Some TE copies may be positioned one after another in tandem organization, and some chromosomal regions have several complete or incomplete copies of a specific TE [65].

In contrast to the aforementioned karyological revolution found in the *Austrolebias* genus group, our previous cytogenetic studies in individuals of *C. melanotaenia* from the same ponds from the Laguna Merin basin showed low karyotypic variation in diploid number, as well as in the large blocks of heterochromatin at subtelomeric regions, while rDNA active regions lacked evidence of major genomic instability [51]. This is consistent with our findings regarding the high content of TEs in *G. charrua* in comparison with the *C. melanotaenia* genome, which also is reflected in a higher rate of duplicate genes in this taxon. Altogether, these cytogenetic and genomic features suggest a different genomic impact linked to the insertion and the increase of elements belonging to some TE superfamilies in the *Austrolebias* genus group compared to *C. melanotaenia*.

### Differences in gene content, structure, patterns of TE insertion, and selective genome constraints among taxa

The fact that *C. melanotaenia* has a highly fragmented assembly and, yet, we were able to recover a number of genes (20,236) similar to that of the other species, suggests that a fragmented genome assembly is not the only factor influencing the high number of genes predicted in *G. charrua*. Interestingly, we found the highest proportion of duplicate genes in this species, opening the possibility of an actual expansion in some gene families and an overall redundancy in gene content. Gene duplication has been proposed as an evolutionary mechanism responsible for neofunctionalization and subfunctionalization phenomena [66]. Unfortunately, the resulting assembly of *G. charrua* was too fragmented to address questions regarding duplicated genes and their functionality. On the other hand, we found shorter exons in *G. charrua*, *N. whitei*, and *C. melanotaenia*. Altogether, these observations seem paradoxical considering that the genomes of these species have a lower proportion of non-repetitive content (i.e. 23,5% in *G. charrua*). However, when restricting the set of orthologs analyzed to single-copy orthologs, we did not observe the same pattern, suggesting that such a feature may be associated with the duplicated gene set.

The analysis of the insertional patterns of TEs in genic regions indicates that approximately one third of annotated genes in *G. charrua* overlap with TE superfamilies, with L2 and TcMar-Tc1 being the most prevalent in congruence with presenting the highest number of TE fragments detected. The general pattern shows that the largest number of genes have a low number of fragments, while very few genes have high numbers of them. At the same time, more genes contain a higher number of fragments of DNA transposons than retrotransposons. Wells and Feschotte [61] propose that the future fate of a TE depends on where it initially inserts in the genome. In this sense, TEs can present little insertional bias, be inserted in genomic regions that minimize their deleterious effects, and/ or target sites that likely facilitate their subsequent propagation. Remarkably, in the present analysis, among other peculiar cases, we found evidence of a past activation event of the Rex-Babar superfamily. For instance, one case which is a unique feature of *G. charrua*, is the very distinguishable peak of activity at values ranging between 30 and 40 Kimura units. Strikingly, 11.79% of the fragments in the peak are located within a single gene that codes for a Leukocyte elastase inhibitor (LEI) also known as Serpin 1B (Supplementary Table 3). This is noteworthy considering that the expression levels of Serpin B2, an orthologue of this gene in humans, have been found to correlate with the expression levels of neighborhooding TEs in lung cells [67].

Distinct niches for different superfamilies of TEs and selection pressure against large insertions in flanking regions and exons have been described in other taxa [56, 68]. When comparing the fragment distribution in genic subregions in *G. charrua*, we found L2 to be the most prevalent in almost all of them whereas a higher proportion of the TcMar-Tc1 superfamily members contribute to overall exon length. Therefore, these patterns suggest the possible occurrence of the amplification and shuffling of genic sequences by TEs. Exon shuffling via transposition of LINE or a DNA transposon type (among other mechanisms), could explain the origin of a new gene set, as well as a high level of isoforms per gene (an average of 2.23) and duplicates as was detected in *G. charrua* (data not shown). According to Bariah et al. [69, among other authors, the insertion of a TE into a gene might result in the origin of new isoforms induced by exonization, truncation, alternative splicing, or the domestication of TE-derived coding sequences into host genes, potentially altering gene function]. Several studies have demonstrated the impact and range of this shuffling and cycling of genomic content on the evolution of plant and animal genomes [49, 56].

Under our present analysis, *G. charrua* is the species with the highest number of tandem repeat regions, presenting shorter repetitions than the other analyzed species. Among 7,286 genes containing tandem repeats longer than 500 bp, 93 of them were cataloged as “highly tandem”. Among them, after functional enrichment analysis, the “exosome multienzyme ribonuclease complex” was identified, which constitutes the most versatile RNA-degradation machine in eukaryotes. When ranking by contribution to gene length, two categories were detected. The category “intracellular anatomical structures” could be associated with the repetitive nature of structural genes such as myosin and actin and, in a sense more specific to annual fishes, to their possible role in maintaining cell integrity during the diapause, a developmentally-programed dormancy state. Secondly, the “transcription cis-regulatory region binding” category was also identified. Cis-regulatory networks coordinate the transcription of multiple genes that function in concert to coordinate entire pathways and complex biological processes. There is now great evidence that TEs have been a rich source of material for the modulation of eukaryotic gene expression [53, 70, 71]. Future transcriptomic and proteomic analyses could clarify the multiple functions of the tandem repeats and the TE patterns of insertion detected in present analysis. As proposed by Nishihara (2019) [11], multidisciplinary studies, including computational genomics, chromosomal analyses, cell biology technology and developmental studies, should be a powerful approach for revealing the multifaceted contributions of a number of TEs to the evolution of genome in a wide variety of groups.

The aforementioned analyses could indicate that, in *G. charrua,* selective constraints have yielded a compact genome structure, preventing TEs insertion within the majority of genes, and showing special and tolerated insertional patterns in another minor fraction of ones. Remarkably, when assessing the degree of the intensity (or relaxation) of selection in a set of single-copy ortholog genes, all analyses were conclusive supporting a stronger purifying selection in *G. charrua* than in the other analyzed taxa, which showed a weaker intensified selection. These results are in contrast to those reported by Cui et al. (2019) in two genera of annual African killifish (*Nothobranchius* and *Callopanchax)* in which they found relatively more genes under relaxed selection than under intensified selection in relation to other non-annual fish species analyzed. Our analyses do not support that relaxed selection is a common feature related to the annual life cycle. Moreover, the present analyses are not concordant with the hypothesis proposed by Cui et al. (2019) for which genome expansion in species with an annual life cycle is likely due to a genome-wide weakening of selective constraints. These authors postulate that annual killifishes display increased mutational rates both in nuclear and mitochondrial genomes due to relaxation of selection consistent with their size expansion [23]. This hypothesis is not aligned with the data presented here, which shows stronger purifying selection in a set of single-copy ortholog genes, nor with the analysis of the dynamics of superfamily expansion aforementioned in *G. charrua*, as well as with previous results in the mitochondrial genome of this species [72]. Importantly, our analysis does not rule out the possibility that relaxed selection plays a role in the evolution of annualism in African killifishes. Rather, it challenges the broad generalization that relaxed selection is inherently associated with an annual life cycle. It is also important to acknowledge that, due to the fragmented nature of the *G. charrua* genome assembly, our selection analysis excluded duplicated genes. Therefore, we cannot rule out the possibility that relaxed selection may be acting on those duplicated loci.

As the *Austrolebias* genus group genome is highly unstable as described above, all present data could indicate that the large genome size in *G. charrua* could be under high compartmentalization, presenting a high proportion of compact genes as the result of intensified selection. Our data could be suggesting that a large fraction of TEs are predominantly clustered in gene-poor niches (perhaps heterochromatic and/or highly dynamic NOR regions, as mentioned above), indicating strong purifying selection against insertions near coding sequences or as a consequence of insertion site preferences in a few cases (i.e. Serpin 1B gene). It is known that the insertion of TEs directly into coding sequences may simply disrupt the functioning of the gene [73].

The hypothesis of compartmentalization of niches with high TE density and niches mostly composed of genes has been previously proposed by many authors in different taxa [48, 53]. Natural selection, genetic drift, and population structure are also powerful factors shaping the distribution and accumulation of TEs, facilitating future propagation while mitigating deleterious effects on host cell function [53].

### Burst of TE activity and hypothetical historical scenarios for genome expansion in the *Austrolebias* genus group

Our results suggest that the very recent (11–12 Mya) genome amplification suffered by killifish of the *Austrolebias* genus group is likely an ongoing process that began with the birth of this clade and may be the result of its recent life history and the requirements of inhabiting a highly variable and challenging environment in a temperate Neotropical region of the Pampas. Given that the last common ancestor of the *Austrolebias* genus group and *C. melanotaenia* existed ~ 25 Myr ago [3, 45], the massive genome-wide changes have occurred between that time and the emergence of the ancestral *Austrolebias* genus group*s* taxa as all members of the genus display a similar, expanded, genome size [7].

Belyayev proposed a hypothetical scenario for genome expansion mediated by bursts of TE amplification preceding speciation in small marginal populations [74]. This explanation provides a plausible basis for genome and chromosome evolution in this annual killifish genus inhabiting temporally heterogeneous environments in South America. Indeed, outbreeding rate has been shown to correlate with the diversity of TEs [75]. Under the influence of unusual and unpredictable ecological (abiotic) conditions, TEs become active; their mobilization produces genetic variation, epigenetic alterations, and high rates of karyotypic reorganization, including changes in the species-specific chromosomal pattern. Since the discovery of TEs, Barbara McClintock (1984), and subsequently other authors [73, 76], have suggested their potential to modulate host gene expression networks in response to specific environmental stress.

On the other hand, another possible source of endogenous genomic stress could be the results of the hybridization between species leading to several genome reorganizations to be driven by TEs [76, 77]. Earlier, McClintock (1984) proposed the genomic shock hypothesis in which hybridization between two species constitutes a source of stress that could disrupt the control mechanisms of TEs causing their activation [73]. Romero-Soriano et al. [78] described the mobilization of at least 28 TEs in hybrids between *Drosophila buzzatii* and *Drosophila koepferae*. In this sense, the most recent findings concerning the ancient hybrid zone between *G. charrua* and its sister taxon *G. reicherti* have corroborated the occurrence of natural hybridization among populations of the genus. This could suggest the possible involvement of these events promoting bursts of TEs in the genomes of hypothetical ancestral populations of the group. Stressful conditions that release constraints and promote the activity burst of TEs are likely, considering that these species undergo extreme departures from normal developmental programs (diapauses), as well as presenting population dynamics characterized by isolation and admixture at times of flooding, yielding to interpopulation hybridization events and the consequent genomic shock [6, 79].

It is impossible to ignore that, if, as we theorize, lifestyle and environment are key factors in the genome size expansion observed in the Austrolebias genus group, then why do we not observe 3 Gb genomes in *N. whitei* and in *C. melanotaenia*? Both species share similar lifestyles and habitats with *G. charrua*. Indeed, *C. melanotaenia* and *G. charrua* usually coexist within the same ponds. One possibility may be related to the different genomic backgrounds of these species, a reflection of the distinct geographic origin of their ancestral populations [9, 45].

Finally, it is interesting to note that—although to a lesser degree—*C. melanotaenia* and *N. whitei* have expanded their genome sizes as well, opening the possibility of other more ancestral TE amplification events in the common ancestor between *G. charrua*, *C. melanotaenia*, and *N. whitei.*

## Conclusions

The genome of *Garcialebias charrua* represents an extreme case among diploid actinopterygian fishes, with a size three times larger than that of its closest relatives and dominated by transposable elements (TEs). Our results indicate that this expansion is driven by a recent and massive amplification of multiple TE superfamilies—particularly LINEs and DNA transposons—that together account for the unusually high proportion of repetitive sequences and the genomic instability of this species.

Rather than being randomly distributed, TEs in *G. charrua* frequently insert within gene regions, suggesting a role in duplication, sequence reshuffling, and the emergence of novel regulatory elements. This may have produced a genomic landscape with an apparently elevated number of genes and isoforms, although part of this signal could reflect assembly artifacts due to the high content of repetitive sequences. Importantly, the subset of single-copy orthologs supports the view that genes in *G. charrua* are under stronger purifying selection compared with other killifishes, in contrast to the relaxed selection reported in Old World annual lineages.

The combined effects of TE activity, possible gene duplication, and selective filtering provide a plausible explanation for the remarkable chromosomal diversity and adaptive potential of this genus in highly variable Neotropical environments. Taken together, our findings highlight *G. charrua* as a valuable model for understanding how bursts of TE activity can reconfigure genomes and contribute to evolutionary diversification in vertebrates.

## Supplementary Information

Supplementary file 1.

## Data Availability

The genome assemblies generated and analysed during the current study are available in the NCBI repository under the PRJNA956886 accession number. Other datasets generated are available in the Zenodo repository (10.5281/zenodo.14435859), or included in this published article [and its supplementary information files]. In case they are not, they will be available from the corresponding author on request. Several of the results resulting from this work rely on scripts and pipelines developed during this research, all of which have been organized into a single repository to facilitate and standardize the analysis of TEs in genomic contexts. We called this software “TE-workflows” and it is available on GitHub (https://github.com/fgajardoe/TE-workflows).
