# Supplementary material for: A recent and rapid genome expansion driven by the amplification of transposable elements in the Neotropical annual killifish Garcialebias charrua
Source: Biol Res. 2025 Nov 27;59:1. doi: 10.1186/s40659-025-00649-8 (PMC12763893; doi:10.1186/s40659-025-00649-8)
Supplement: Supplementary file 1 [file 40659_2025_649_MOESM1_ESM.pdf]

A recent and rapid genome expansion driven by the amplification of transposable elements in the Neotropical annual killifish Garcialebias charrua

Gajardo F, Valdivieso C, Di Genova A, Pereiro L, Arezo MJ, Nardocci G, Rojas N, Gutiérrez V, Papa NG, Berois N, Orellana A, Gutiérrez RA, González M, Mendez M, Montecino M, Hodar C, Glavic A, Maass A, García G, Allende ML.

Supplementary Information I

Index

Figure S1: Phylogenetic tree of representative TEs from the L2 superfamily across species..3
Figure S2: Phylogenetic tree of representative TEs from the Rex-Babar superfamily across species.............................. 4
Figure S3: Phylogenetic tree of representative TEs from the RTE-BovB superfamily across species.............................. 4
Figure S4: Phylogenetic tree of representative TEs from the TcMar-Tc1 superfamily across species................................5
Figure S5: Phylogenetic tree of representative TEs from the hAT-Ac superfamily across species....................................5
Figure S6: Phylogenetic tree of representative TEs from the hAT-Charlie superfamily across species............................. 6
Figure S7: Distribution of Ka/Ks ratios across species in genes with significant results according to RELAX................ 7
Figure S8: Statistical significance in all-vs-all comparison of Ka/Ks distribution for genes under intensified selection.. 7
Figure S9: Patterns of insertion of TEs within genes in the Garcialebias charrua genome..8
Figure S10: Patterns of insertion of TEs within genes in the Nematolebias whitei genome... 8
Figure S11: Patterns of insertion of TEs within genes in the Cynopoecilus melanotaenia genome................................... 9
Figure S12: Patterns of insertion of TEs within genes in the Austrofundulus limnaeus genome....................................... 9
Figure S13: Patterns of insertion of TEs within genes in the Kryptolebias marmoratus genome.................................... 10
Figure S14: Patterns of insertion of TEs within genes in the Nothobranchius furzeri genome.. 10
Figure S15: Patterns of insertion of TEs within genes in the Oryzias latipes genome...11
Figure S16: Tandem period size distribution across species considering the most abundant TE superfamilies together.. 11
Figure S17: Tandem period size distribution across species considering the most abundant TE superfamilies separately.. 12
Table S1. Sequencing metrics for the genomes of *G. charrua* and *C. melanotaenia*..13
Table S2. Gene annotations metrics for the subset of single-copy genes identified by BUSCO....................................... 13
Table S3. Summary of the repetitive elements annotated for each species..13

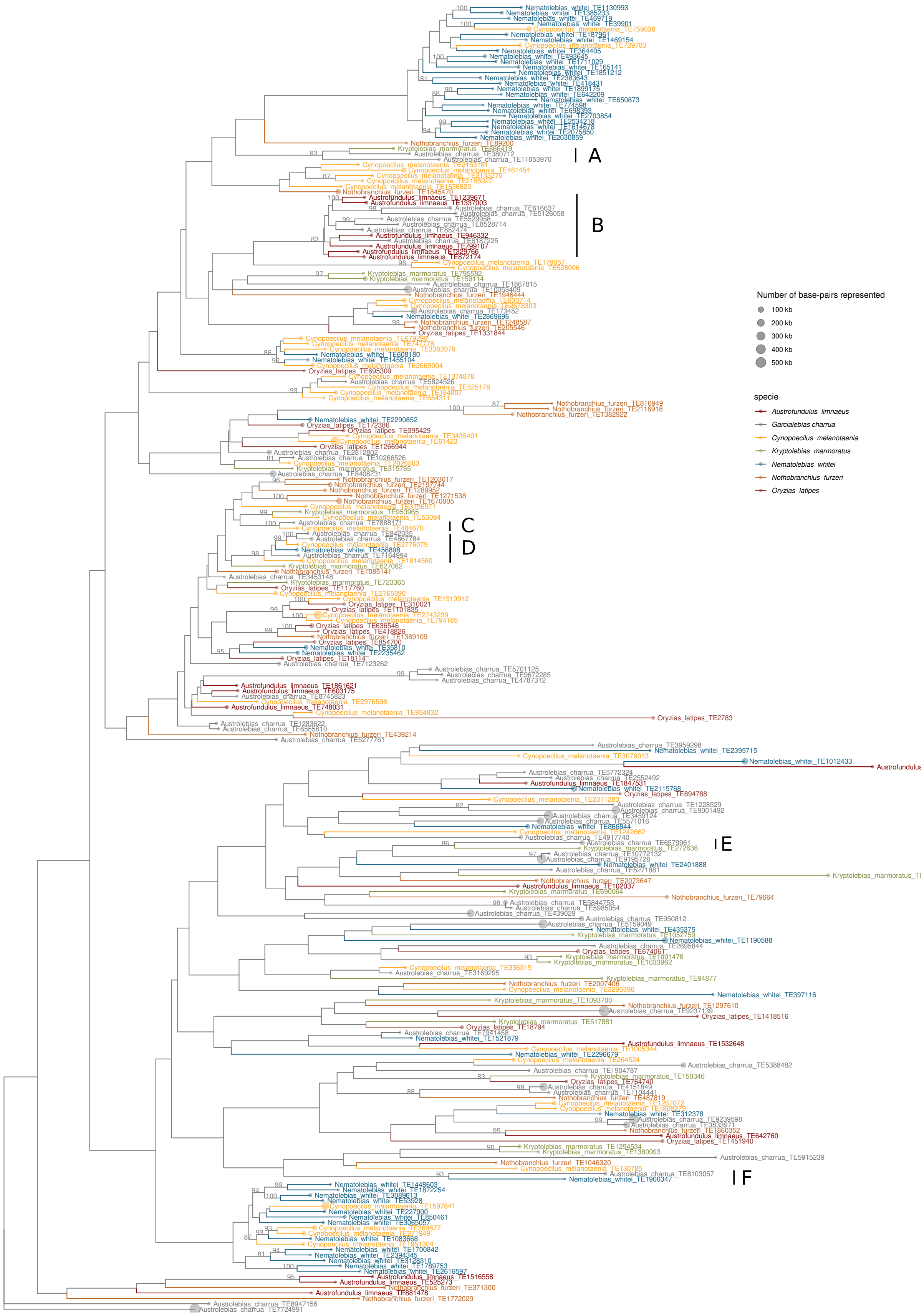

Figure S1: Phylogenetic tree of representative TEs from the L2 superfamily across species.

Maximum likelihood tree based on sequences of representative TEs (centroids) for the L2 superfamily with bootstrap support obtained from 100 replicates. Support values above 80 are shown. Edges are colored by species. The circle size is proportional to the number of fragments of TEs in the genome that share sequence similarity with the representative TE. Note that tip labels display the old species name *Austrolebias charrua* instead of the recently adopted *Garcialebias charrua*.

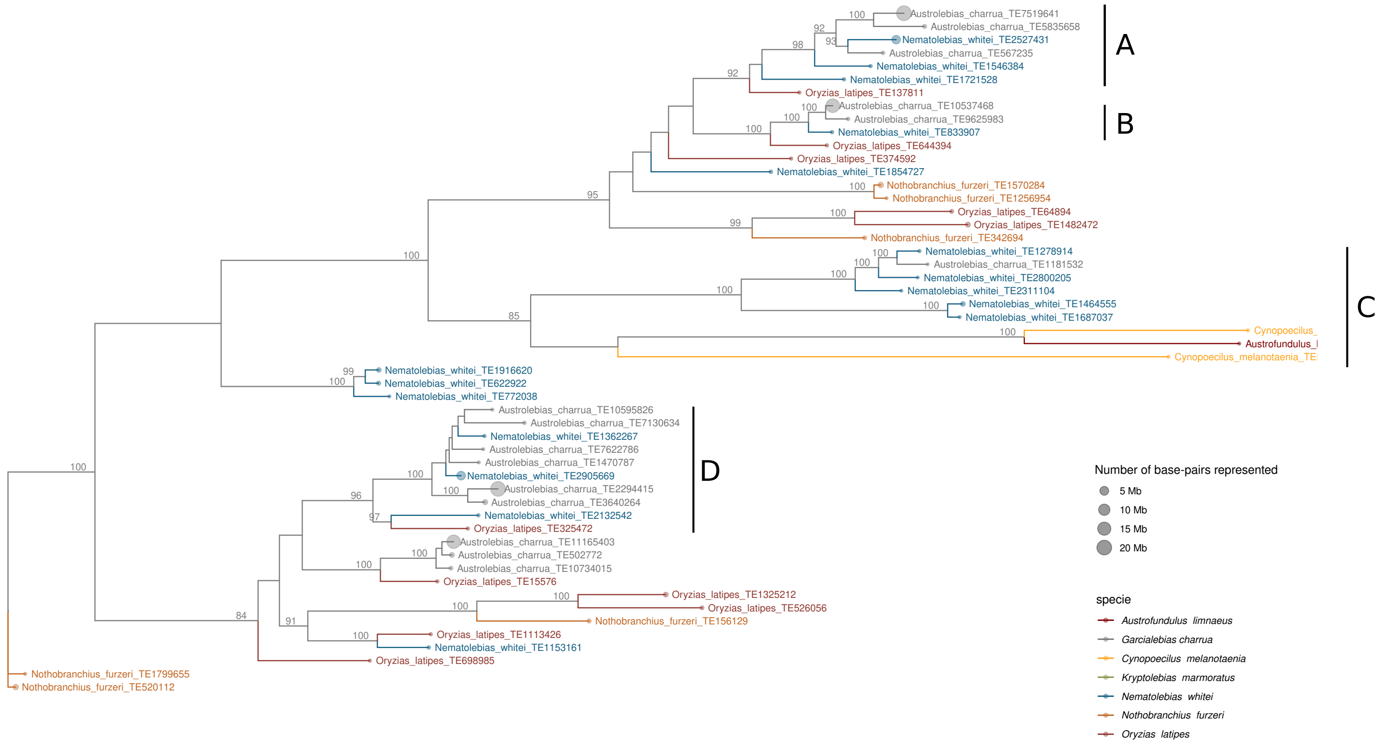

Figure S2: Phylogenetic tree of representative TEs from the Rex-Babar superfamily across species. Maximum likelihood tree based on sequences of representative TEs (centroids) for the Rex-Babar superfamily with bootstrap support obtained from 100 replicates. Support values above 80 are shown. Edges are colored by species. The circle size is proportional to the number of fragments of TEs in the genome that share sequence

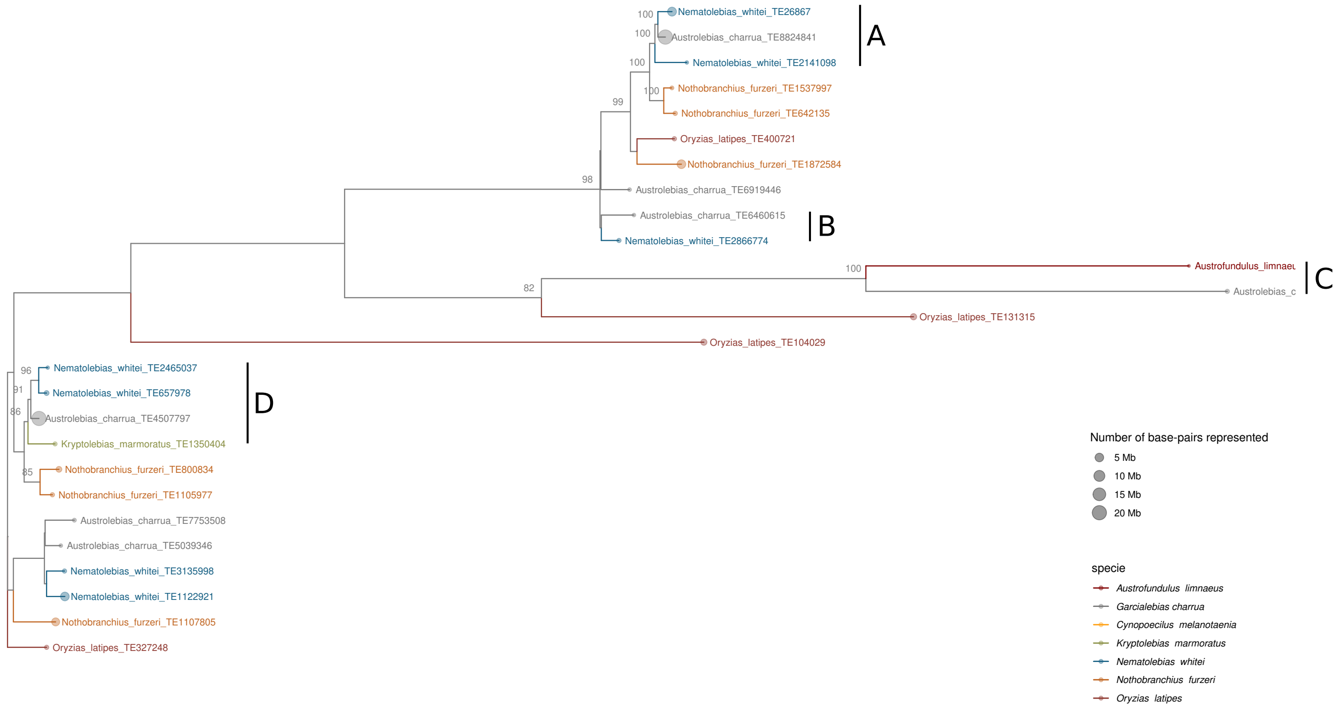

Figure S3: Phylogenetic tree of representative TEs from the RTE-BovB superfamily across species. Maximum likelihood tree based on sequences of representative TEs (centroids) for the RTE-BovB superfamily with bootstrap support obtained from 100 replicates. Support values above 80 are shown. Edges are colored by species. The circle size is proportional to the number of fragments of TEs in the genome that share sequence similarity with the representative TE.

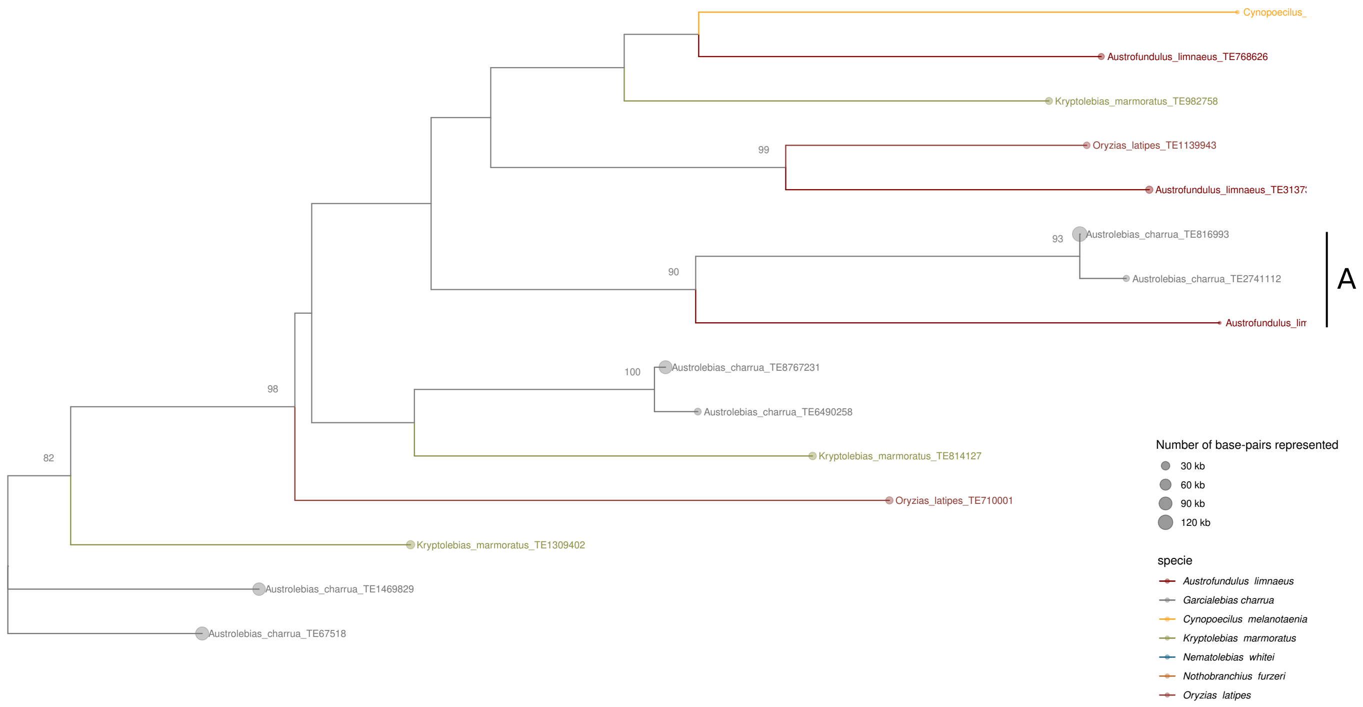

Figure S4: Phylogenetic tree of representative TEs from the TcMar-Tc1 superfamily across species.

Maximum likelihood tree based on sequences of representative TEs (centroids) for the TcMar-Tc1 superfamily with bootstrap support obtained from 100 replicates. Support values above 80 are shown. Edges are colored by species. The circle size is proportional to the number of fragments of TEs in the genome that share sequence

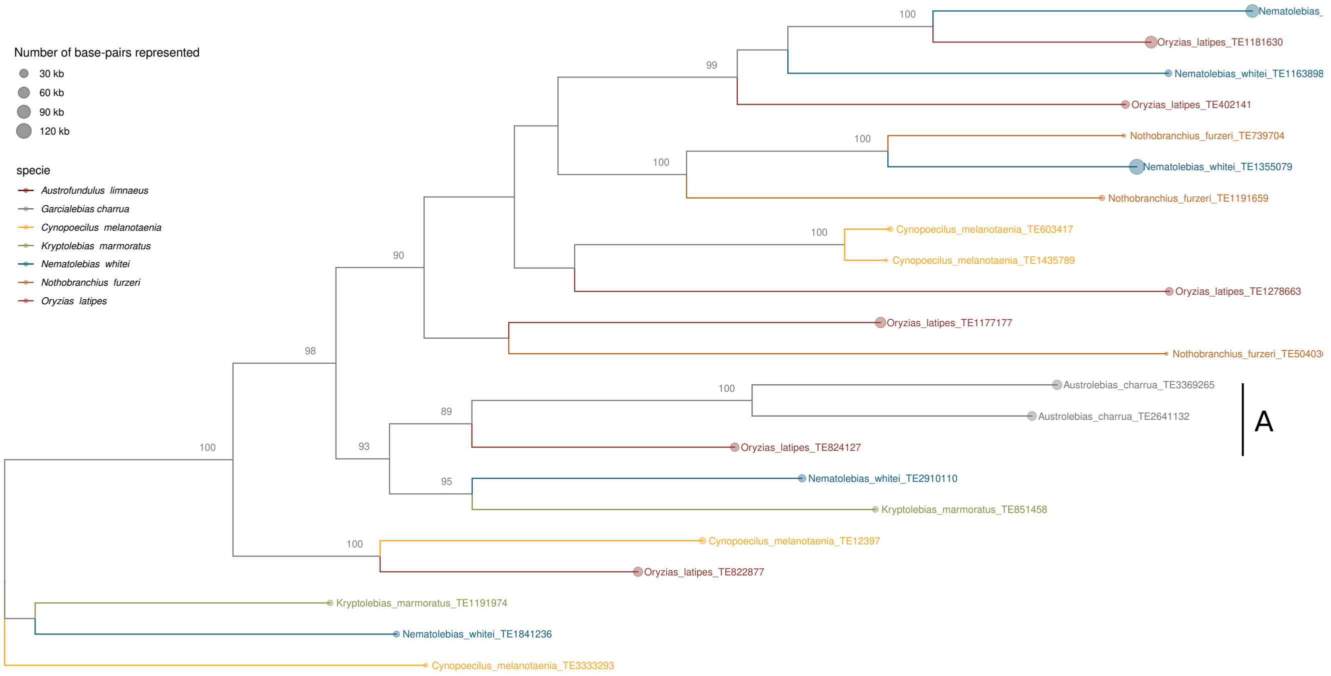

Figure S5: Phylogenetic tree of representative TEs from the hAT-Ac superfamily across species.

Maximum likelihood tree based on sequences of representative TEs (centroids) for the hAT-Ac superfamily with bootstrap support obtained from 100 replicates. Support values above 80 are shown. Edges are colored by species. The circle size is proportional to the number of fragments of TEs in the genome that share sequence similarity with the representative TE.

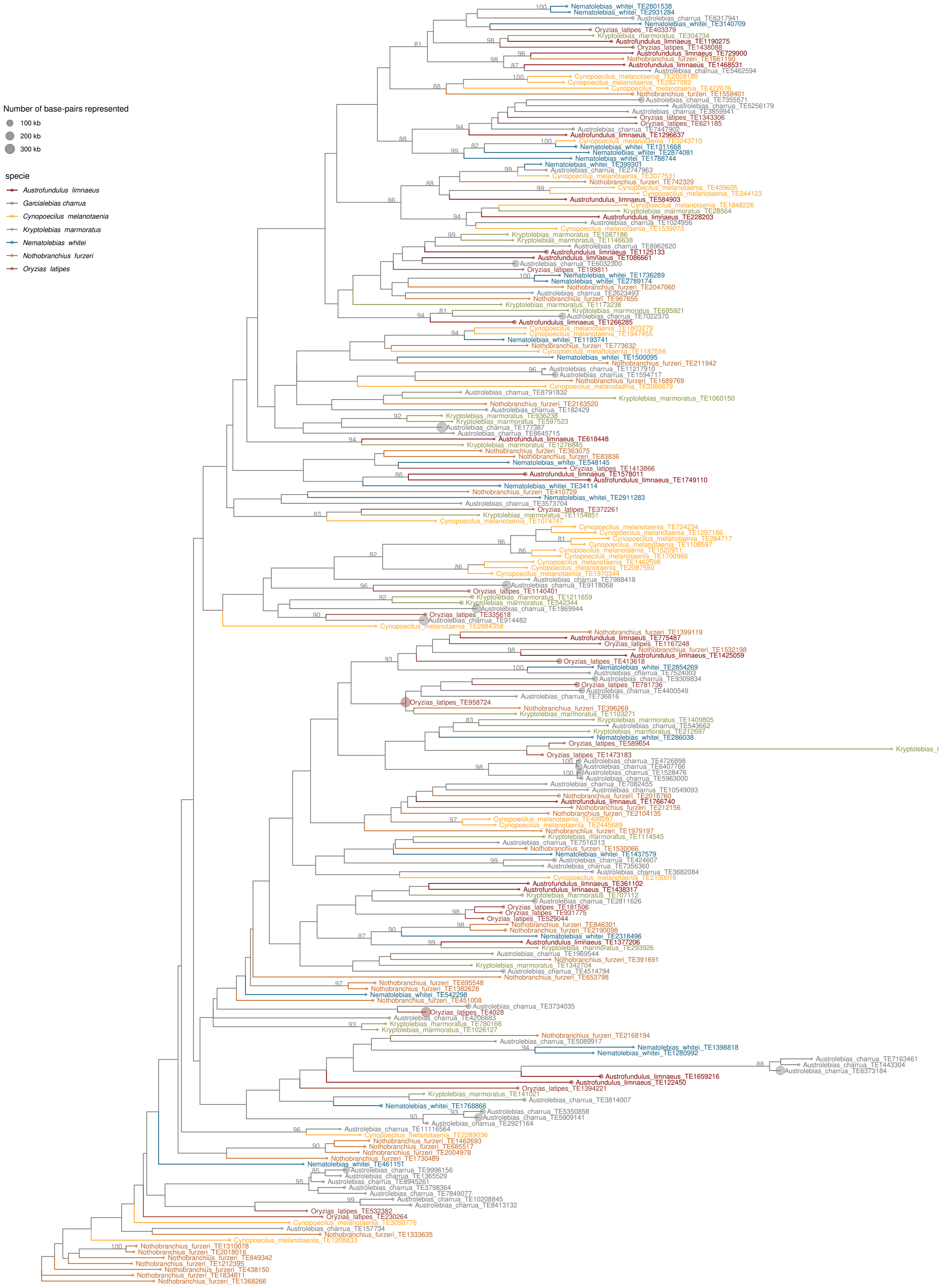

Figure S6: Phylogenetic tree of representative TEs from the hAT-Charlie superfamily across species. Maximum likelihood tree based on sequences of representative TEs (centroids) for the hAT-Charlie superfamily with bootstrap support obtained from 100 replicates. Support values above 80 are shown. Edges are colored by species. The circle size is proportional to the number of fragments of TEs in the genome that share sequence similarity with the representative TE.

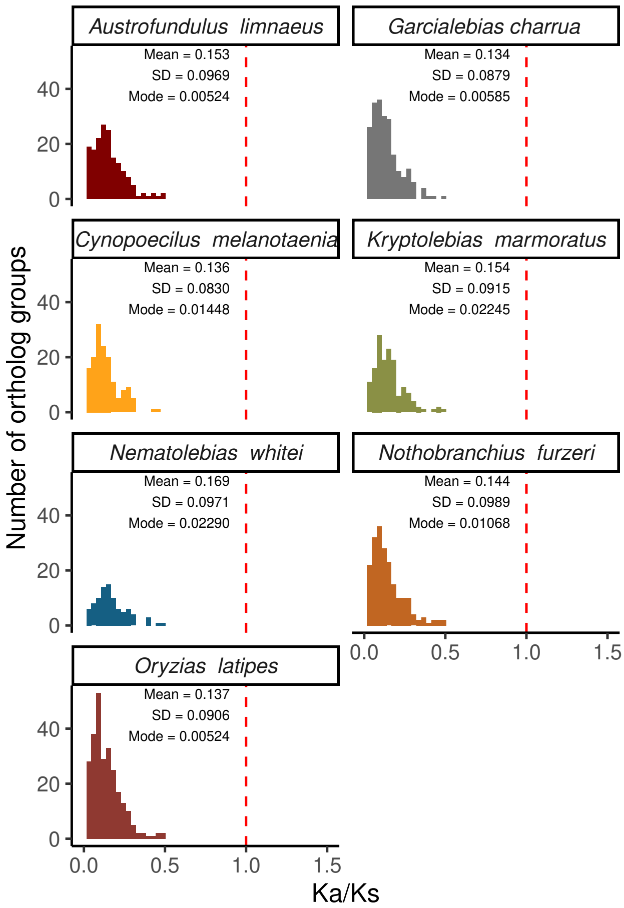

Figure S7: Distribution of Ka/Ks ratios across species in genes with significant results according to RELAX analysis.

Genes were considered irrespective of the type of selection operating on them (intensified or relaxed). The red line points to neutral selection.

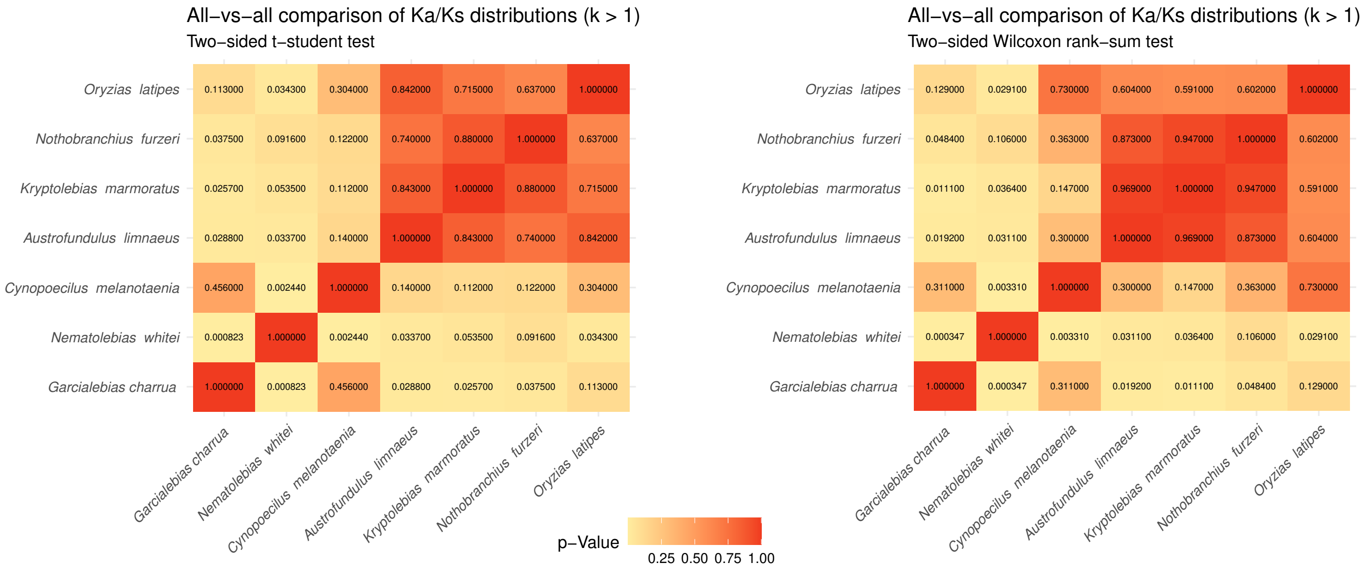

Figure S8: Statistical significance in all-vs-all comparison of Ka/Ks distribution for genes under intensified selection.

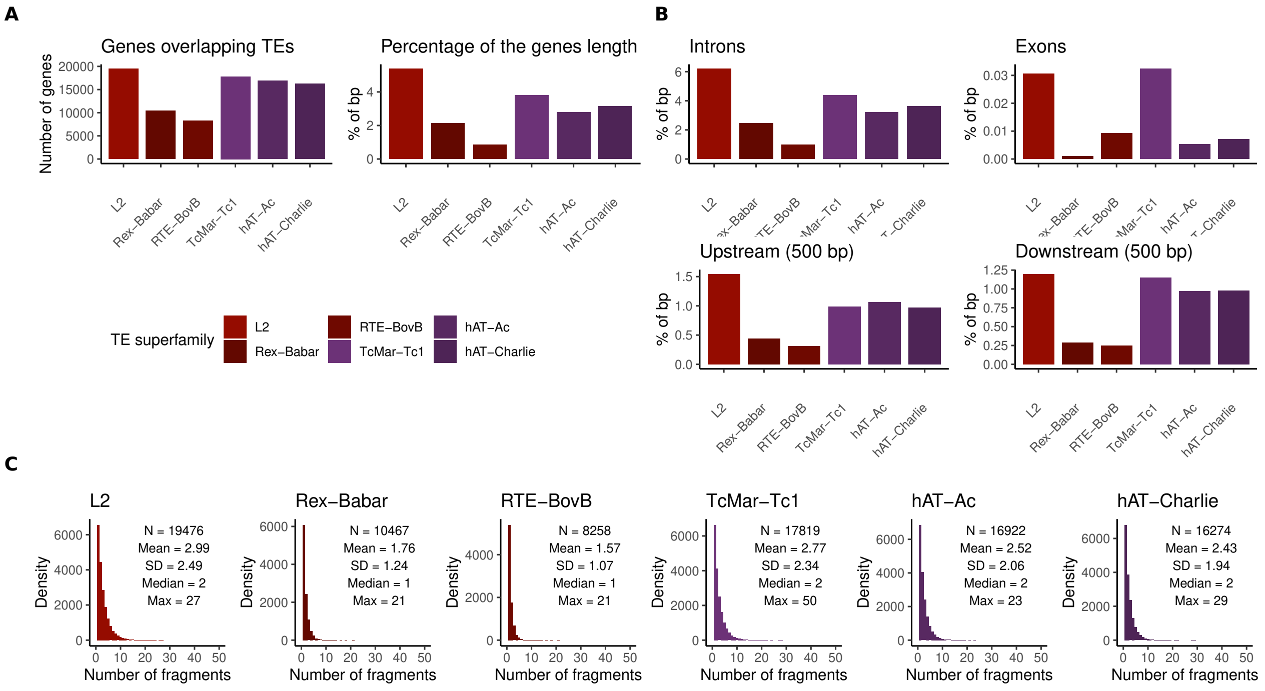

Figure S9: Patterns of insertion of TEs within genes in the *Garcialebias charrua* genome.

(A) TE-derived tandem repeats of the most abundant TE superfamilies overlapping genes and their respective contribution to the gene length.

(B) TE-derived tandem repeats of the most abundant TE superfamilies overlapping gene subregions.

(C) Distribution of the number of TE fragments on genes for the most abundant TE superfamilies.

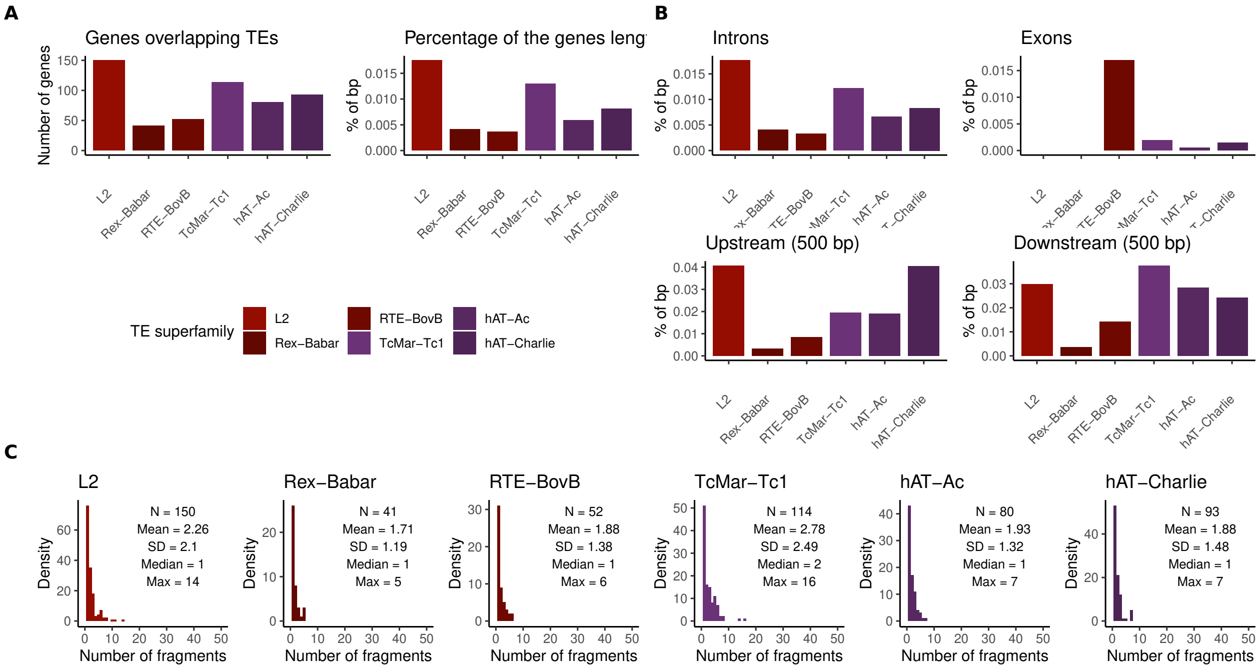

Figure S10: Patterns of insertion of TEs within genes in the *Nematolebias whitei* genome.

(A) TE-derived tandem repeats of the most abundant TE superfamilies overlapping genes and their respective contribution to the gene length.

(B) TE-derived tandem repeats of the most abundant TE superfamilies overlapping gene subregions.

(C) Distribution of the number of TE fragments on genes for the most abundant TE superfamilies.

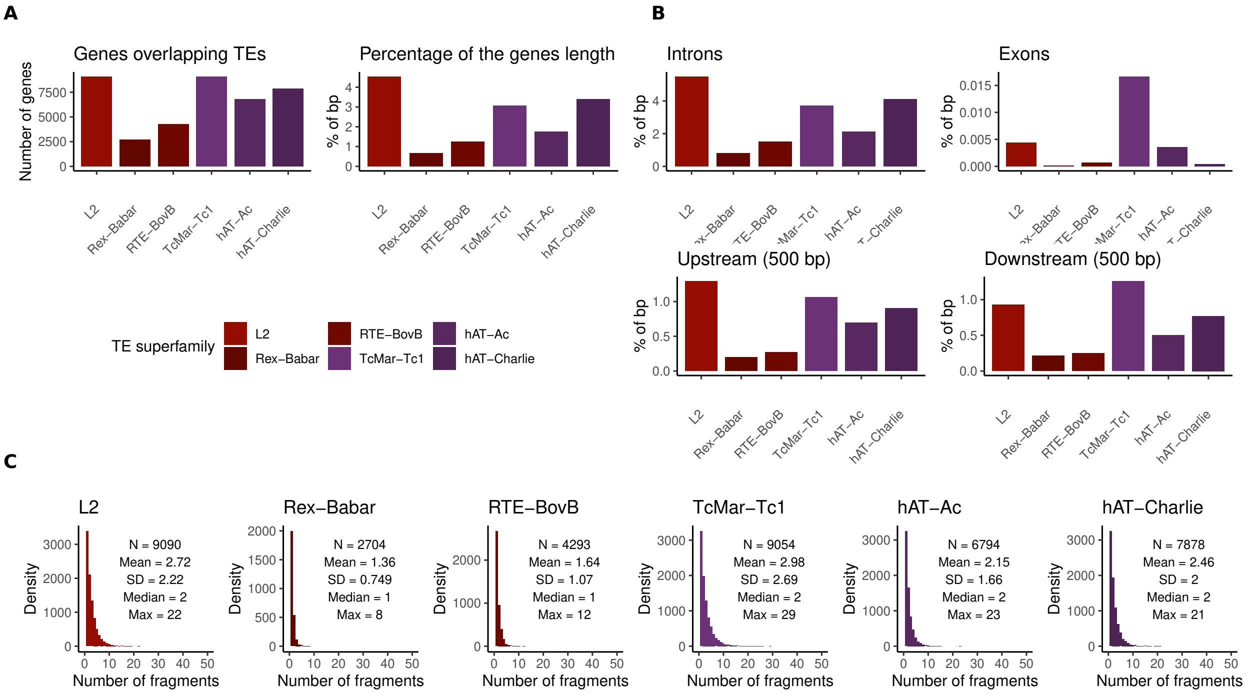

Figure S11: Patterns of insertion of TEs within genes in the *Cynopoecilus melanotaenia* genome.

(A) TE-derived tandem repeats of the most abundant TE superfamilies overlapping genes and their respective contribution to the gene length.

(B) TE-derived tandem repeats of the most abundant TE superfamilies overlapping gene subregions.

(C) Distribution of the number of TE fragments on genes for the most abundant TE superfamilies.

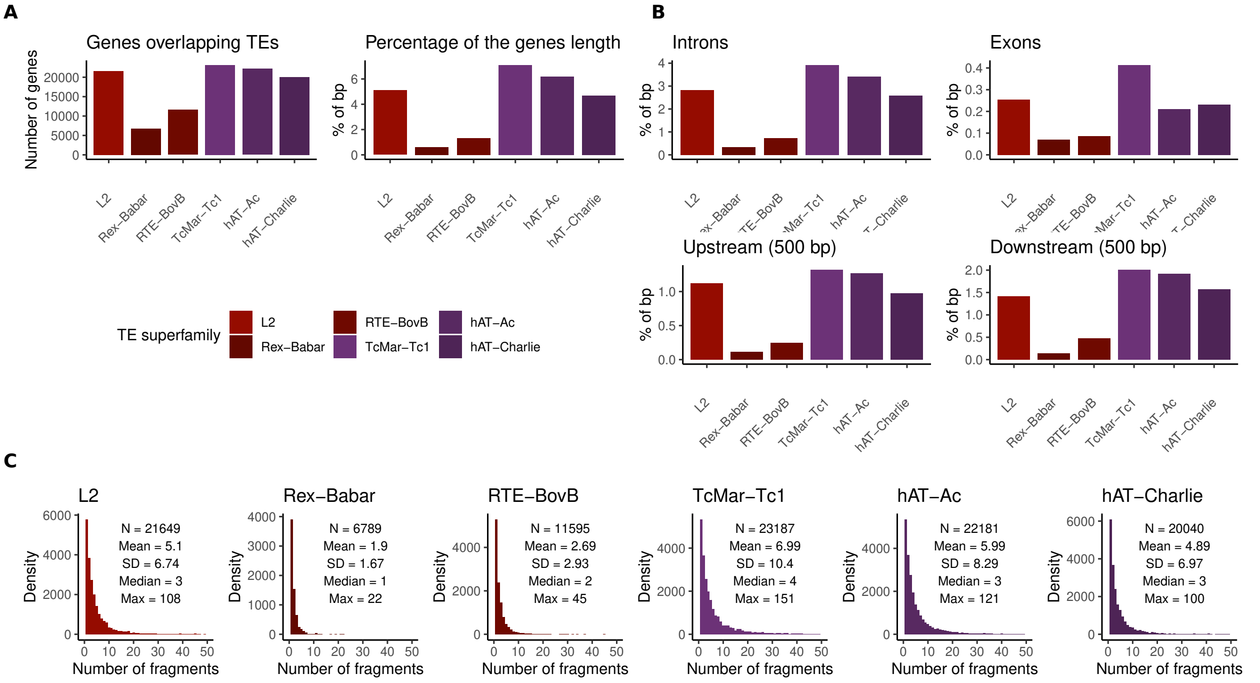

Figure S12: Patterns of insertion of TEs within genes in the *Austrofundulus limnaeus* genome.

(A) TE-derived tandem repeats of the most abundant TE superfamilies overlapping genes and their respective contribution to the gene length.

(B) TE-derived tandem repeats of the most abundant TE superfamilies overlapping gene subregions.

(C) Distribution of the number of TE fragments on genes for the most abundant TE superfamilies.

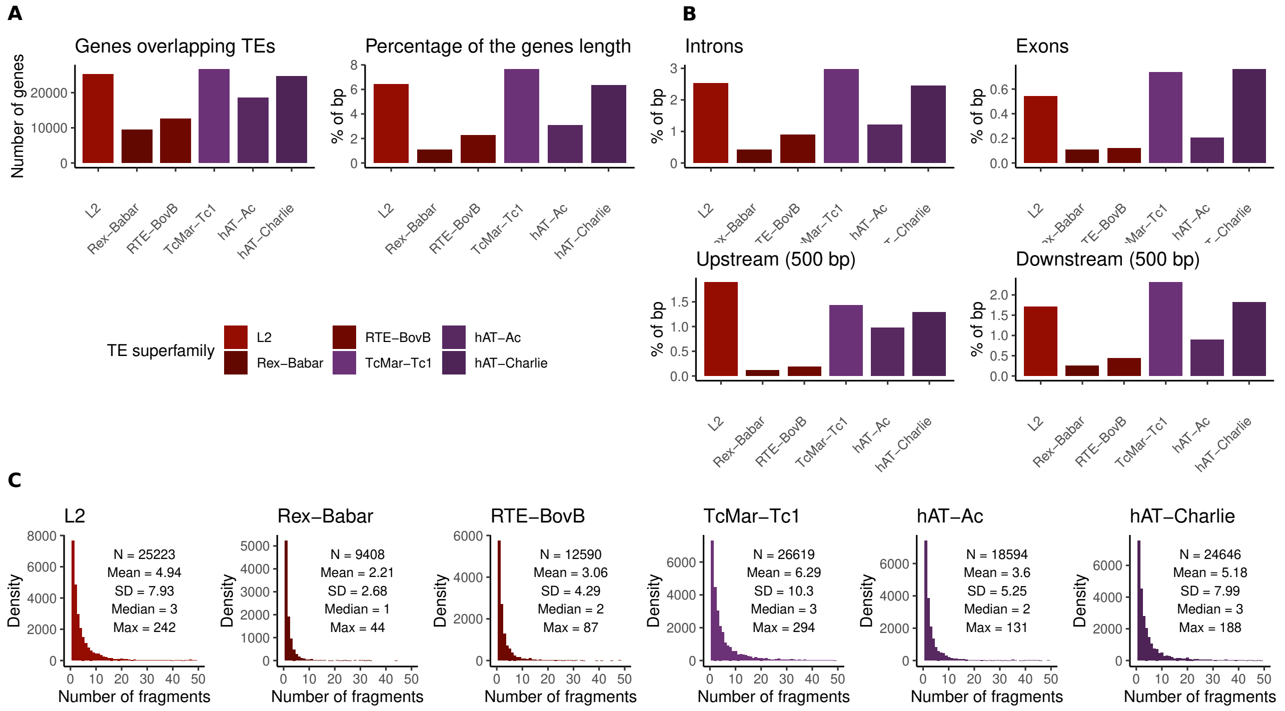

Figure S13: Patterns of insertion of TEs within genes in the *Kryptolebias marmoratus* genome.

(A) TE-derived tandem repeats of the most abundant TE superfamilies overlapping genes and their respective contribution to the gene length.

(B) TE-derived tandem repeats of the most abundant TE superfamilies overlapping gene subregions.

(C) Distribution of the number of TE fragments on genes for the most abundant TE superfamilies.

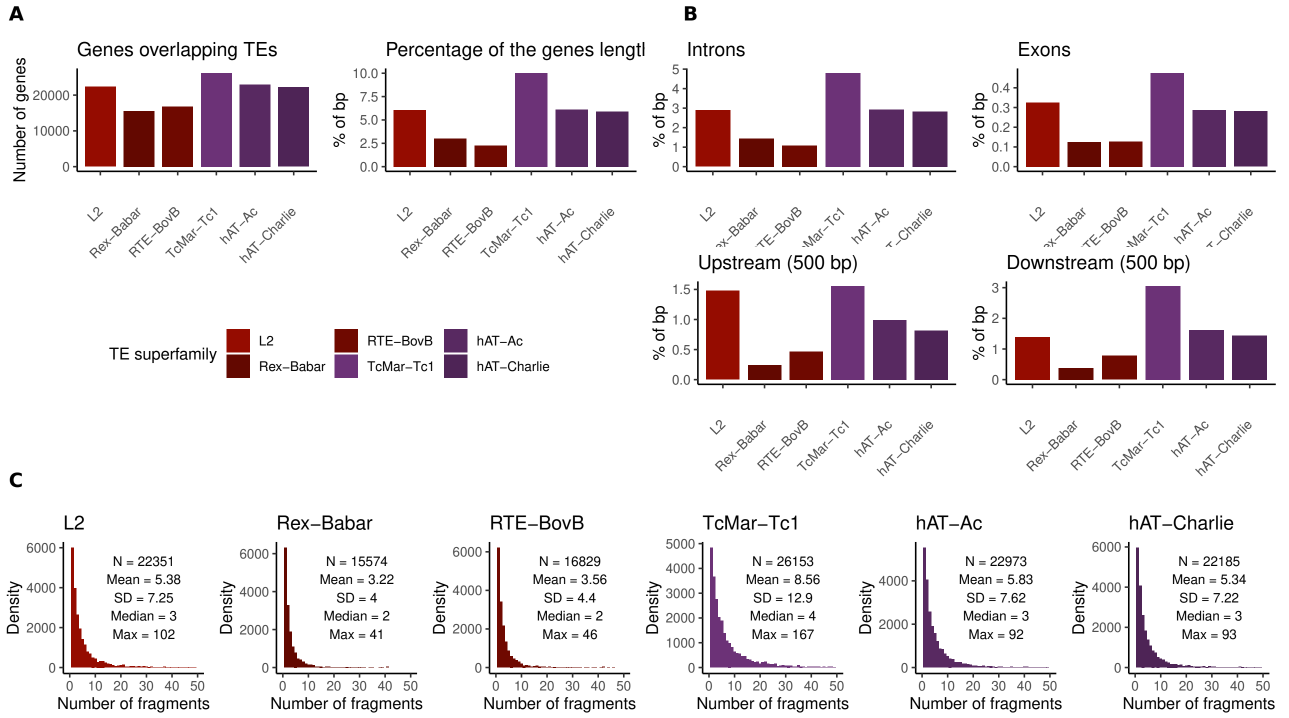

Figure S14: Patterns of insertion of TEs within genes in the *Nothobranchius furzeri* genome.

(A) TE-derived tandem repeats of the most abundant TE superfamilies overlapping genes and their respective contribution to the gene length.

(B) TE-derived tandem repeats of the most abundant TE superfamilies overlapping gene subregions.

(C) Distribution of the number of TE fragments on genes for the most abundant TE superfamilies.

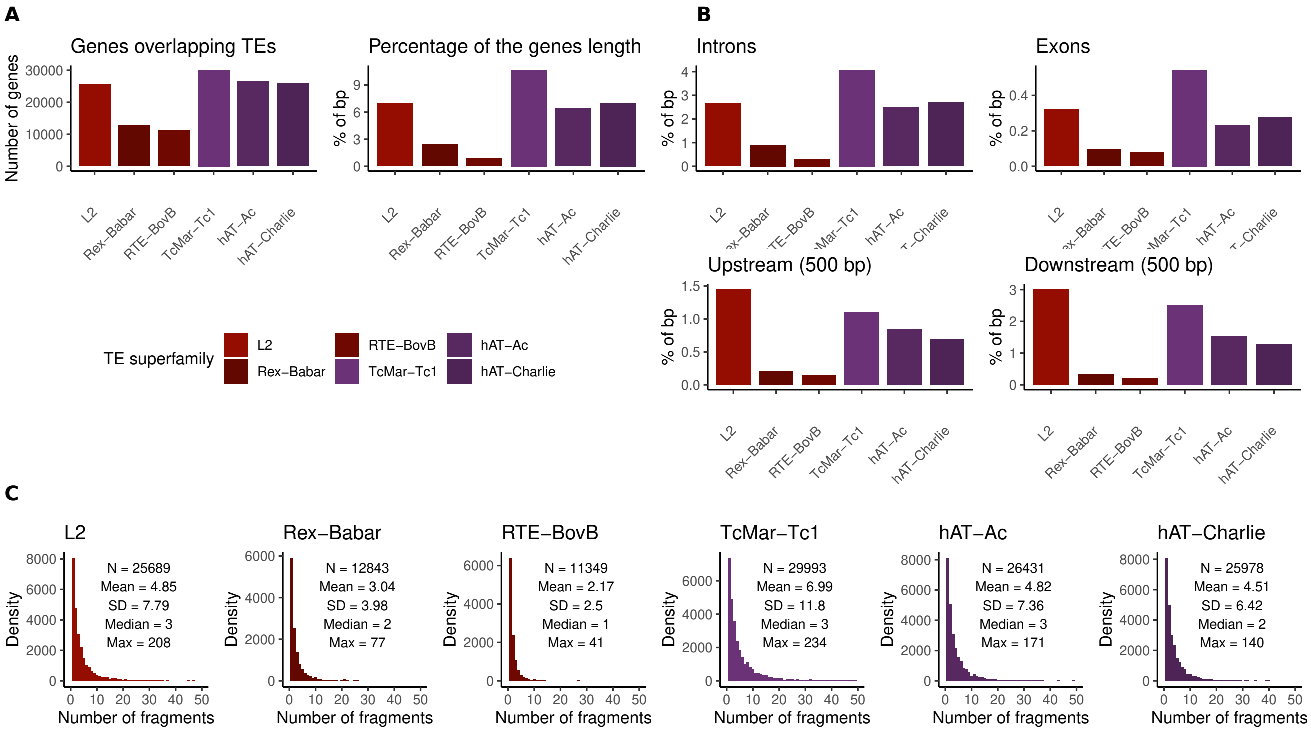

Figure S15: Patterns of insertion of TEs within genes in the *Oryzias latipes* genome.

(A) TE-derived tandem repeats of the most abundant TE superfamilies overlapping genes and their respective contribution to the gene length.

(B) TE-derived tandem repeats of the most abundant TE superfamilies overlapping gene subregions.

(C) Distribution of the number of TE fragments on genes for the most abundant TE superfamilies.

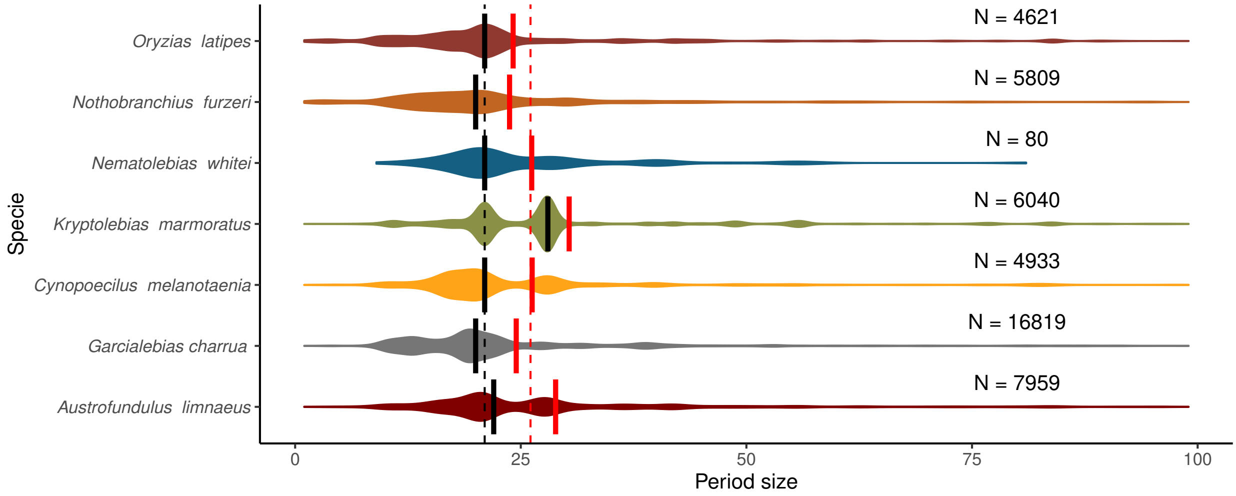

Figure S16: Tandem period size distribution across species considering the most abundant TE superfamilies together.

The black crossbar points to the median tandem size of each species; the red crossbar points to the mean tandem size of each species; The black dashed line corresponds to the median of the median tandem size considering all species; The red dashed line corresponds to the mean of the mean tandem size considering all species.

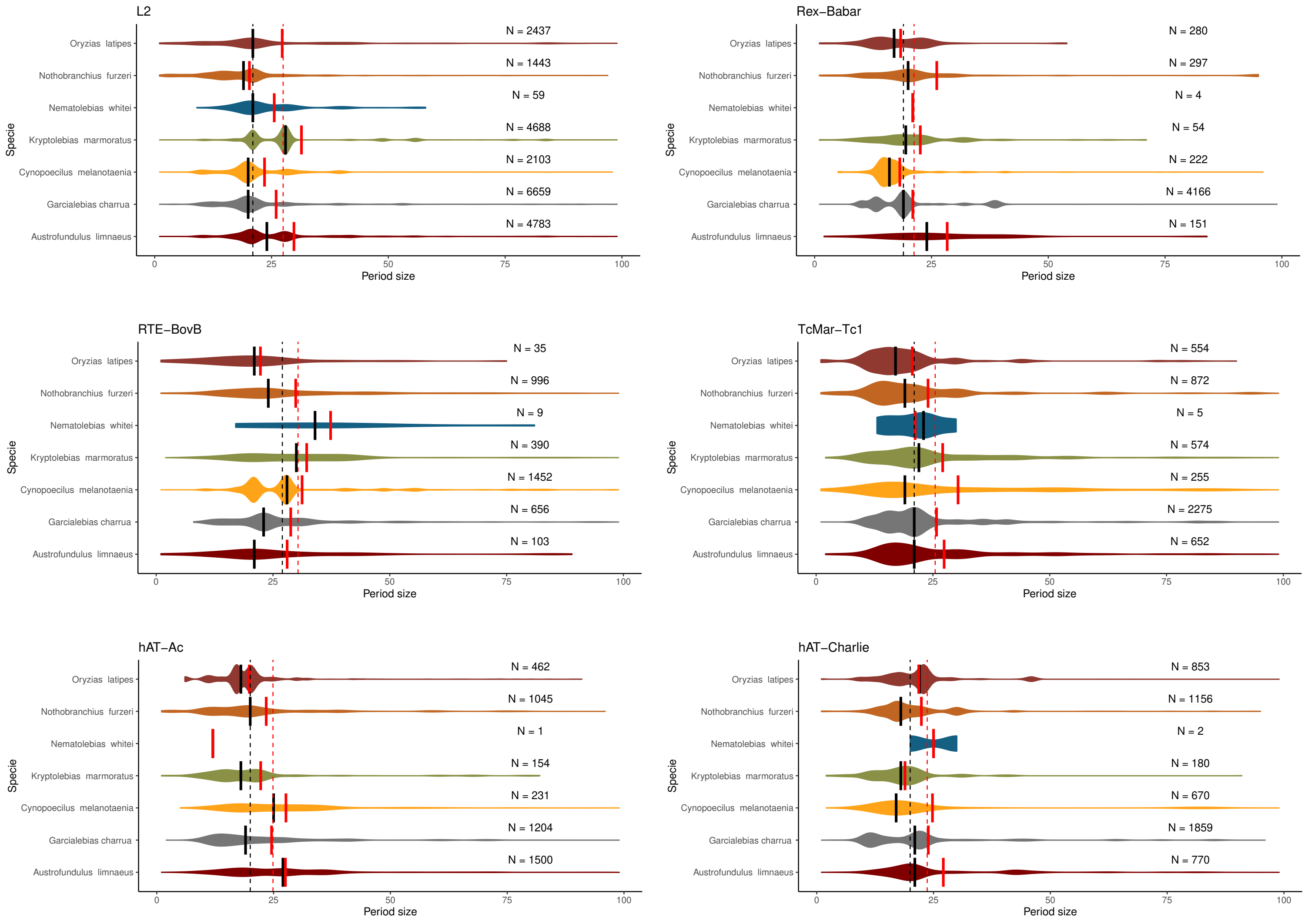

Figure S17: Tandem period size distribution across species considering the most abundant TE superfamilies separately.

The black crossbar points to the median tandem size of each species; the red crossbar points to the mean tandem size of each species; The black dashed line corresponds to the median of the median tandem size considering all species; The red dashed line corresponds to the mean of the mean tandem size considering all species.

Supplementary Tables

Table S1. Sequencing metrics for the genomes of *G. charrua* and *C. melanotaenia.*

Species	Library description	Sequencing Technology	Total Bases	Read count	Genome Coverage
G. charrua	Overlapping paired-ends with a fragment size of 280 bp.	Illumina	39,265,491,870	260,036,370	13.1
G. charrua	Mate-paired with 5 Kb insert size.	Illumina	39,016,677,694	258,388,594	13.0
G. charrua	Overlapping paired-ends with a fragment size of 280 bp.	Illumina	97,873,334,780	324,083,890	32.6
G. charrua	Long reads with a average length of 11 Kb.	PacBio	19,673,087,511	1,766,893	6.6
C. melanotaenia	Overlapping paired-ends with a fragment size of 280 bp.	Illumina	94,074,164,468	374,797,468	94.1

Table S2. Gene annotations metrics for the subset of single-copy genes identified by BUSCO.

	G. charrua	*N. whitei*	*C. melanotaenia*	*A. limnaeus*	*K. marmoratus*	*N. furzeri*	*O. latipes*
Number of genes	2,020	4,414	2,886	4,467	4,774	4,706	4,702
Number of exons	13,492	35,661	20,319	35,897	39,221	37,977	37,965
Number of introns	11,472	31,248	17,433	31,431	34,448	33,273	33,265
Avg. exon length	193.10	183.35	190.70	184.53	183.61	183.87	184.71
Avg. intron length	1,443.59	2,123.95	895.42	1,572.74	1,234.81	1,701.85	1,327.79
Avg. number of exons per gene	6.68	8.08	7.04	8.04	8.22	8.07	8.07
Avg. number of introns per gene	5.68	7.08	6.04	7.04	7.22	7.07	7.07

Table S3. Summary of the repetitive elements annotated for each species.

	G. charrua	*N. whitei*	*C. melanotaenia*	*A. limnaeus*	*K. marmoratus*	*N. furzeri*	*O. latipes*
**Number of families**	3,510	2,446	2,430	2,739	2,024	2,938	2,223
**Number of distinct superfamilies**	63	46	50	59	52	51	53
Classification							
**DNA**	1,819	1,299	1,290	1,645	1,160	1,660	1,230
**LINE**	1,235	898	954	936	708	1,040	819
**LTR**	314	153	83	78	87	127	110
**RC**	7	13	10	14	21	7	4
**SINE**	36	1	16	9	13	12	10
**Satellite**	1	17	0	0	0	0	0
**tRNA**	2	1	0	0	0	0	0
**Retroposon**	0	1	2	1	0	0	1
**rRNA**	0	0	0	0	0	1	4
**Unknown**	96	63	75	56	35	91	45
